# Monoclonal Antibodies for Chronic Pain Treatment: Present and Future

**DOI:** 10.3390/ijms221910325

**Published:** 2021-09-25

**Authors:** Eva M. Sánchez-Robles, Rocío Girón, Nancy Paniagua, Carmen Rodríguez-Rivera, David Pascual, Carlos Goicoechea

**Affiliations:** Área de Farmacología, Nutrición y Bromatología, Departamento de Ciencias Básicas de la Salud, Facultad de Ciencias de la Salud, Universidad Rey Juan Carlos, High Performance Research Group in Experimental Pharmacology (PHARMAKOM), Unidad Asociada I+D+i al Instituto de Química Médica (CSIC), Avenida de Atenas s/n, 28922 Alcorcón, Madrid, Spain; eva.sanchez@urjc.es (E.M.S.-R.); nancy.paniagua@urjc.es (N.P.); carmen.rodriguez@urjc.es (C.R.-R.); david.pascual@urjc.es (D.P.); carlos.goicoechea@urjc.es (C.G.)

**Keywords:** monoclonal antibodies, chronic pain, preclinical, clinical, review

## Abstract

Chronic pain remains a major problem worldwide, despite the availability of various non-pharmacological and pharmacological treatment options. Therefore, new analgesics with novel mechanisms of action are needed. Monoclonal antibodies (mAbs) are directed against specific, targeted molecules involved in pain signaling and processing pathways that look to be very effective and promising as a novel therapy in pain management. Thus, there are mAbs against tumor necrosis factor (TNF), nerve growth factor (NGF), calcitonin gene-related peptide (CGRP), or interleukin-6 (IL-6), among others, which are already recommended in the treatment of chronic pain conditions such as osteoarthritis, chronic lower back pain, migraine, or rheumatoid arthritis that are under preclinical research. This narrative review summarizes the preclinical and clinical evidence supporting the use of these agents in the treatment of chronic pain.

## 1. Monoclonal Antibodies

Antibodies (Abs) are glycoproteins belonging to the immunoglobulin (Ig) superfamily that are secreted by B cells to identify and neutralize foreign organisms or antigens. Abs comprise two heavy and two light chains and are grouped into different isotypes depending on which type of heavy chain they contain [1].

**Figure 1:**
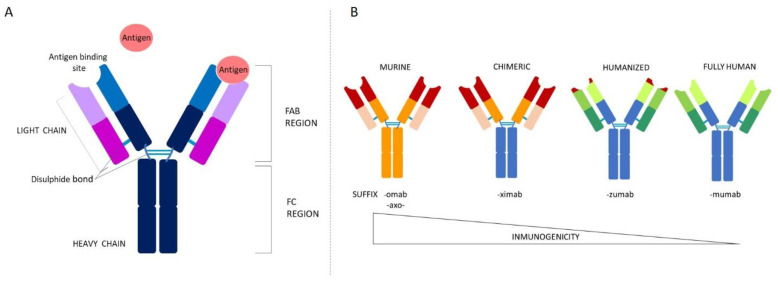
Structure and classification of monoclonal antibodies. (**A**) General structure of mAbs. (**B**) Classification and lexicon of mAbs according to the immunogenicity and their synthetic process. Depicted in warm colors are the murine origin portions of the antibody, and in blue and green human are origin segments.

In the late quarter of the past century, monoclonal antibodies (mAbs) were synthetically created with therapeutic purposes. They are typically derived from the γ-immunoglobulin (or IgG) isotype, and share a common structure based on two heavy chains and two light chains connected by inter chain–disulphide bonds forming a Y-shaped structure (Figure 1A). The hypervariable regions of each heavy and light chain combine to form the antigen binding site, referred to as the fragment antigen binding domain (Fab), while the crystallizable or constant fragment (Fc) domain responsible for effector function is composed of two constant domains [1,2].

mAb are produced by cloning a unique B cell. All subsequent Abs derived from these clones can be traced back to a unique parent cell. Traditionally, the earliest Abs were created by immunizing experimental animals with an antigen with subsequent purification of the serum to isolate the Ab fraction [2,3].

### 1.1. Classification and Types of mAbs

According to their origin and the dictation of the WHO [4], there are four types of mAbs: murine, chimeric, humanized, and human [5,6] (Figure 1B).

- Murine: this was the first mAb discovered and reproduced. This type of mAb emerges from a collection of B lymphocytes from the spleen of a mouse, which are then fused with an immortal myeloma cell line. All these mAbs are identified with a name that ends in -omab (e.g., muromonab-CD3, capromab). They are often associated with allergic reactions and the induction of anti-drug antibodies (ADAs) [5,7]. Hybrid mouse/rat antibodies are denoted by the syllable -axo- (e.g., catumaxomab).
- Chimeric: attempting to overcome the inherent immunogenicity and reduced effector function of murine mAbs in human and chimeric mouse–human Abs were developed. They utilize the murine antigen-specific variable region, but the remaining heavy and light chains are human, resulting in mAbs that are approximately 65% human and 35% murine [1]. These mAbs are identified with names ending in -ximab (e.g., rituximab, infliximab) [6]; they exhibit an extended half-life in humans and show reduced immunogenicity, but the propensity to induce ADAs is still considerable [5].
- Humanized: in humanized Abs, only the hypervariable regions of the light and heavy chains are murine [8]; this results in molecules that are approximately 95% human, decreasing the apparition of ADAs. These mAbs are identified with names ending in -zumab (e.g., trastuzumab, alemtuzumab, tanezumab) [5,6].
- Human: the fully human mAbs are created using animals carrying human Ig genes. These transgenes include parts of the variable regions that enable the recombination of the human Abs [5,9]. These mAbs are less antigenic and better tolerated compared to the other classes of mAbs. They are identified with names ending in -umab (e.g., ofatumumab, fulranumab, erenumab) [6].

Similarly, as occurred with generics derived from synthetic drugs, biosimilars have also been introduced in the clinic. The European Medicines Agency (EMA) defines a biosimilar as “a biological medicinal product that contains a version of the active substance of an already authorised original biological medicinal product in the European Economic Area” (European Medicines Agency: Guideline on similar biological medicinal products (2014) [10]). In addition, the importance of biosimilar Abs to those already in the market is outstanding; due to the intrinsic variability of all biologicals and the manufacturing process of these products, a biosimilar cannot be considered an identical copy of the originally approved biological product [11]. Small differences between the biosimilar and the reference product are allowed, but it is necessary to demonstrate that these differences are not clinically significant. “Similarity to the reference medicinal product in terms of quality characteristics, biological activity, safety and efficacy based on a comprehensive comparability exercise needs to be established” [10].

It must be considered that several differences, from a pharmacological point of view, can be observed when comparing classical drugs with mAbs (Table 1).

### 1.2. Mechanisms of Action and Clinical Applications of mAbs

**Figure 2:**
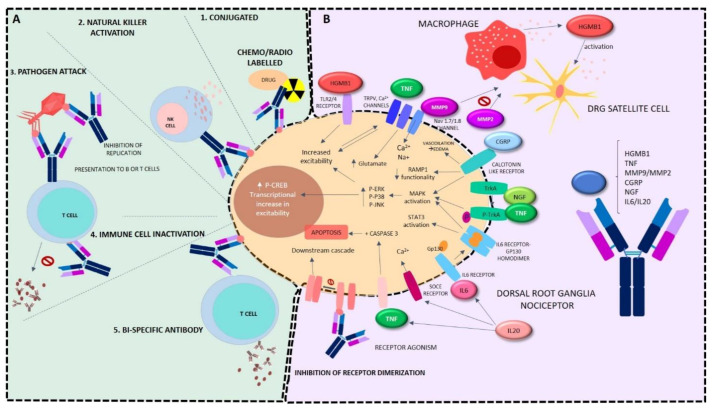
Mechanism of action of monoclonal antibodies. Potential targets of mAbs according to the mechanism of action and signaling cascade pathways modified by them. (**A**) Depicting their importance as (**1**) conjugated with radio or chemoligands for oncological treatments; (**2**) activators of natural killer cells; (**3**) antiviral agents, inhibiting viral proliferation or presenting pathogens to T or B cells; (**4**) immunosuppressors being tremendously relevant in autoimmune diseases; (**5**) dual role players as bi-specific antibodies binding two cells and generating response in them. (**B**) Focusing on the treatment of pain, aiming at the inhibition of nociceptive pathways. Clockwise, they may recognize HGMB preventing its biding to TLR2/4 receptors, and its release by macrophages and subsequent activation of other satellite cells. They may be designed to bind TNF, hence blocking the increased excitability of neurons it produces when it binds to TRPV and calcium channels, also preventing the phosphorylation of Trk-A and caspase 3-mediated apoptosis. Regarding stalling MMPs, a success as an inhibition of the opening of sodium channels may be attained, as well as the blocking of the activation of macrophages and other satellite cells MMPs are able to carry out. Targeting CGRP, the block of the activation of the calcitonin receptor may be obtained. Similarly, as with TNF, when designed to bind NGF, an inhibition of the phosphorylation of Trk-A is achieved. Interleukin 6 renders a dimerization of its receptor together with Gp130, which is blocked by the binding of mAbs designed for such purpose, as it occurs when they bind interleukin 20, where the inhibition of the entrance of calcium ions would take place. Ca: calcium; CGRP: calcitonin gene-related peptide; DRG: dorsal root ganglia; pERK: phosphorylated extracellular signal-regulated kinase; Gp130: glucoprotein 130; HGMB: high mobility box; IL: interleukin-6; IL20: interleukin-20; p-JNK: phosphorylated c-Jun N-terminal kinase; MAPK: mitogen-activated protein kinase; MMP: matrix metalloproteinase; Na: sodium; NGF: nerve growth factor; NK: natural killer; p-P38: phosphorylated protein p38; RAMP1: receptor activity-modifying protein 1; SOCE: store-operated calcium entry; STAT3: signal transducer and activator of transcription 3; TLR4: toll-like receptor- 4; TNF: tumor necrosis factor; Trk-A: tyrosine kinase A; p-Trk-a: phosphorilated tyrosine kinase; TRPV: transient receptor potential cation channel.

In brief, mAbs are designed so that once the Fab region recognizes and binds the antigen, it is directed to a block in the pathological exacerbation that occurs. mAbs, therefore, are of great use for several pathologies. Depending on the disease to be treated, mAbs activate different pathways (Figure 2).

- Autoinmune diseases: these conditions are characterized by a proliferation, migration, and activation of B and T cells, leading to cytokine and proinflammatory molecule secretion, ensuing cellular damage. Herein, mAbs are in charge of suppressing excessive responses, hence inhibiting cellular recruitment or the interaction of antigen-presenting cells with T cells and blocking the activation and depletion of B and T cells inhibiting the release of proinflammatory cytokines [12].
- Infectious diseases: mAbs may bind viruses, directly inhibiting their replication, or, as in the case of HIV, by binding to CD4 receptors of immune cells, impeding the entrance of the virus inside the host cell [12].
- Oncology: mAbs herein attempt to inhibit tumorigenesis and tumor cell migration through several pathways. The first approach is tumor cell killing. Firstly, conjugated mAbs work as specific treatment carriers to the tumor, herein including drug-conjugated Ab (carrying the drug itself) or radioactively conjugated Ab (carrying radiation to the tumoral cell) [13]. The second approach is by receptor binding, by induction of cellular apoptosis cascade through receptor agonism, or by antagonism-specific receptors inhibiting receptor dimerization or ligand binding, rendering downstream cascades that lead to reduced proliferation or apoptosis.
- Moreover, another strategy resides in the driving of the immune cells to the tumorigenic cells for them to carry out the assault. This process may be carried out by phagocytosis, antibody-dependent cellular cytotoxicity using immune cell effectors such as natural killers, complement mediated cytotoxicity, or by inhibiting the checkpoint escape (inactivation of T cell inhibitory receptors). Tumor cells bind through PD-L1 to PD-1 receptors of T cells to inhibit immune response that would destroy malignant proliferation; hence, the binding of mAbs to PD1 and PD-L1 blocks this inhibition [14]. Moreover, carcinogenic cells may be specifically targeted by vascular and stromal cell ablation by precise toxin delivery, inhibition of agonists in the vasculature, or specific stromal cell inhibition. The last strategy resides in bispecific mAbs, which consist of two arms, with one arm recognizing cancer cells and the other activating antigens on immune effector cells including CD3 [15].

## 2. Chronic Pain and Possible Usefulness of mAbs in Its Treatment

Pain is described “an unpleasant sensory and emotional experience associated with, or resembling that associated with, actual or potential tissue damage” [16]. This definition, however, does not differentiate between the so-called “acute pain” (responsible for informing the body of the presence of a danger) and chronic pain that becomes a disease, losing its function of signaling a danger and producing suffering for the patient [17]. Classically, pain has been classified as nociceptive (the cause and the stimulus producing pain can be detected and, very often, localized) and neuropathic (caused by an injury or disease of the somatosensory nervous system) [17]. In both cases, inflammation can contribute to the physiopathology of pain. However, chronic pain is often mixed, with nociceptive and neuropathic components. Frequently, acute pain can progress to chronic pain through multiple peripheral and central mechanisms. In chronic pain, pathophysiological changes can completely alter the nociceptive system, including increased excitability of nociceptors; changes in dorsal root ganglia, spinal cord, and glial cells; and modifications in inhibitory control and downward modulation [18,19], as well as immune-to-nervous system interactions [19,20].

Central and peripheral sensitization are characterized by an amplified response to the noxious stimuli. These changes result in alterations in the sensorial perceptions, leading to increased pain sensation to a noxious stimuli (hyperalgesia) as well as painful responses to non-noxious stimuli (allodynia) [19].

Actually, there are numerous pharmacological options for the treatment of chronic pain such as non-steroidal anti-inflammatory drugs (NSAIDs), opioids, and some antidepressants such as serotonin and norepinephrine reuptake inhibitors (SNRIs) or gabapentinoids [21]. However, it is necessary to search for new therapies due to the limitations of current treatments, such as the lack of efficacy or the undesirable adverse effects.

Despite the type of pain (nociceptive or neuropathic), the role of inflammation in chronification and sensitization is clearly demonstrated [22]. Several pro-inflammatory mediators are involved in neuropathic pain. Amongst others, eicosanoids, bradykinin, neurotrophins, and several cytokines stand out, revealing a close link between inflammation and neural hypersensitivity [17,19] and offering theorical targets for the development of new mAbs.

In the last decade, biologic therapies have been investigated in the treatment of chronic pain, and several mAbs have emerged as attractive alternatives to conventional analgesics, providing some potential benefits: high affinity and specificity for predetermined ligands or targets in pain transmission and neurogenic inflammation that may lead to the absence of unwanted adverse effects. As mentioned in Table 1, the long half-lives of mAbs mean that doses are administered less frequently (monthly or quarterly dosing), which makes the patient tolerate and adheres better to the treatment. Moreover, its metabolism is through the reticuloendothelial system, with low hepatic or renal toxicity, and due to their molecular characteristics, they have a high limited ability to cross the blood–brain barrier, reducing the possibility of CNS adverse events. However, certain disadvantages have also been identified in the use of mAb in the treatment of pain: due to their high molecular size, hydrophilicity and gastric degradation, and parenteral administration (intravenous, intramuscular, or subcutaneous routes) is necessary [17,22,23]. Moreover, it is highly relevant that mAbs do not cross the BBB. Since the role played by CNS in pain is highly determinant, this can be considered as an important limitation. In that sense, maybe a combination therapy of mAbs targeting peripheral pain components and more conventional centrally penetrant drugs would be more appropriate for pain management than the single therapy.

The application of mAbs in the treatment of pain is justified and based on the large number of specific targets involved in the transmission of pain and that contribute to its chronification; some of them are described below and can be seen schematically in Figure 2.

Mitogen-activated protein kinase (MAPK) activation is a key pathway in neuropathic pain consolidation, sensitization, and inflammatory mediation [24]. Herein, inactivation of pleiotrophic cytokines such as tumor necrosis factor (TNF), epidermal growth factor receptor, and nerve growth factor (NGF) are key targets for mAbs in the treatment of pain. These peptides activate TrkA phosphorylation and subsequent MAPK cascade with the succeeding increase of p-ERK, p-P38, and p-JNK, as well as consequent increase in transcriptional (p-CREB rise) and non-transcriptional increases in post-synaptic excitability [25]. These mechanisms play a major role in the generation and maintenance of chronic pain, both nociceptive and neuropathic. Moreover, these peptides are also involved in other nociception pathways such as tumor progression enhanced by the epidermal growth factor receptor, which is part of the tyrosine kinase receptor family, and thus its inhibition targeting by mAbs is very interesting to hamper pain conditions associated with cancer. On the other hand, NGF is involved in peripheral sensitization by enhancing the expression of ion channels and receptors on afferent neurons and enhancing the release of pain mediators such as substance P [26]. Similarly, TNF is also related to peripheral sensitization by inducing caspase 3-mediated apoptosis [27]. Furthermore, it is also able to activate TRPV1, leading to a presynaptic increase in glutamate and a postsynaptic increase in several receptor activities, resulting in an escalation in pain transmission; moreover, it is also able to increase proinflammatory cytokine release in several pathologies. Further related to pain transmission are N-type voltage gated calcium channels and voltage gated sodium channels (Nav 1.7 and 1.8), which are highly expressed in C- and Aδ-fibers; thus, these ion channels can be inhibited by mAbs to decrease painful transmission. Also regarding sodium channels, matrix metalloproteinases (MMPs) must be bared in mind, especially MMP9 that, on one hand, acts directly on primary afferent neurons, evoking the expression of the Nav 1.7 and Nav1.8 channels [28], increasing nociceptive afferent transmission in the early phase of the neuropathic pain. On the other hand, together with MMP2, it plays important roles in the development and maintenance of neuropathic pain [29,30], neuroinflammation, and peripheral sensitization since it contributes to neuroinflammation by increasing the activity of satellite glial cells and macrophages, inducing the release of proinflammatory cytokines and chemotaxis [29].

Likewise, in relation to receptor inhibition, calcitonin gene-related peptide (CGRP) is a neuropeptide that is produced by neurons in the central nervous system (CNS) and peripheral nervous system (PNS) and binds to calcitonin-like receptors; it activates MAPK cascade and also modifies the receptor activity-modifying protein 1 (RAMP1) activity, reducing its functionality; consequently, it is key for nociceptive transmission together with substance P and serotonin receptors. Therefore, targeting either CGRP or its receptor, the downstream cascade can be reduced, rendering a decrease in pain evolution [31].

In a similar manner, interleukins such as IL-6 and IL-20, as well as their receptors, may be pursued by mAbs, bearing in mind the importance they have when overexpressed. Both cytokines are key players in the inflammatory component of pain pathogenesis and in peripheral and central sensitization, resulting in increased pain transmission [32]. To block IL activity by means of mAbs could modify the process of peripheral sensitization and also the development of central sensitization induced by this increase in peripheral activity. Moreover, IL-6, by binding to its receptor, is able to promote the homodimerization together with gp130, leading to an activation of the MAPK and the STAT3 pathways [33]. On the other hand, IL-20 is also able to increase IL-6, TNF, and IL-1β production by astrocytes and other glial cells, increasing the proinflammatory atmosphere, also increasing store-operated calcium entry (SOCE) channel activity, resulting in an increase in Ca^2+^ entrance and following an increase in nerve transmission. Its suppression has been proven to reduce neuropathic pain and to restore intracellular calcium homeostasis [34].

Also concerning inflammation, high-mobility group box-1 (HMGB1) seems an important target for mAbs. It is a non-histone DNA-binding protein that regulates gene transcription and replication [35]. It plays a critical role in the response of a tissue to inflammation since it is secreted in the form of an damage-associated molecular patterns from activated cells such as macrophages, monocytes, and glial and dendritic cells, and induces the production of chemokines and cytokines, which, in turn, activate immune and glial cells and stimulate neurons [36]. HMGB1 is upregulated in the dorsal horn of the spinal cord and dorsal root ganglia (DRG) neurons in some rodent models of chronic pain [37,38,39,40,41].

Indirectly, peripheral modifications induced by mAbs administration can modify CNS physiology. In the spinal dorsal horn, the extracellular HMGB1 stimulates different receptors, especially toll-like receptors (TLR) TLR2 and TLR4, receptor for advanced glycation end products (RAGE) and C-X-C chemokine receptor type 4 (CXCR4) in the immune cells [37,41,42], which are highly involved in chronic pain as their activation increases the expression of proinflammatory mediators such as cytokines, chemokines (IL-1β, TNF, monocyte chemotactic protein, and IL-6), and nitric oxide synthetase. Moreover, increased cytokines, such as TNF and IL-1β, can also induce the release of HMGB1, which develops a feedback inflammatory loop involved in the nociceptive transmission [43]. Additionally, it increases the activity of spinal neurons and the synaptic plasticity mediated be the NMDA receptor [38,44]. In the PNS, when there is nerve tissue injury, HMGB1 diffuses from the damaged cell and is critical in the maintenance of neuropathic pain.

Apart from the possible future targets described above, some mAbs have already been developed for the treatment of diverse types of pain or painful conditions.

This review focused on mAbs’ effect on alleviating pain and symptoms that contribute to its relief, not the whole symptoms of the different pathologies. Studies cited in this review were found through Pubmed and ClinicalTrials.gov (accessed on 22 September 2021) searches. The search terms were as follows: “monoclonal antibody”, “chronic pain”, “animal model”, and all pain conditions (“musculoskeletal pain”, “osteoarthritis”, “neuropathic pain”, “rheumatoid arthritis”, “fibromyalgia”, “migraine”, “chronic low back pain”, “cancer pain”, “endometriosis”), and the Boolean operators “AND” and “OR” were used.

A search in https://clinicaltrials.gov (accessed on 22 September 2021) with terms “chronic pain” and “monoclonal antibody” were performed to obtain the controlled clinical trials, observational studies, and the reports derived from clinical studies. Moreover, this search was completed with other published studies, systematic and narrative reviews, meta-analyses, and case studies in Pubmed.

## 3. mAbs in Osteoarthritis Pain

Osteoarthritis (OA) is the most prevalent joint disease, with symptoms affecting 10–12% of the adult population. It is a complex pathology, characterized by articular cartilage damage, low-grade synovial inflammation, and hypertrophic bone changes, leading to chronic pain and functional deterioration [45,46]. OA pain is predominantly nociceptive in origin, although aspects of neuropathic pain may also occur. Peripheral and central sensitization are known to play an important role in this type of pain, and the OA population is likely to be a heterogeneous mix of pain states [47,48].

Traditionally the management of OA has been constrained to symptom relief. The NSAID, acetaminophen, and opioid analgesics are most commonly applied to OA for relieving pain; however, their side-effects often restrict their use [49]. In recent years, there has been substantial progress made in understanding the pathogenesis of OA, and currently, there are emerging treatments targeting inflammation, cartilage metabolism, and subchondral bone remodeling, which may retard the structural progression and induce disease remission [50].

NGF has been implicated in the pathogenesis of OA pain due its ability to facilitate peripheral and central sensitization. The release of several nociceptor-sensitizing inflammatory mediators, including NGF, during cartilage degradation, bone remodeling, and the synovial inflammation process, likely plays an important role in mechanical hyperalgesia in some patients with OA pain [47]. Abs that inhibit the function of NGF and small molecule inhibitors of NGF receptors have been developed and tested in clinical studies to evaluate the efficacy of NGF inhibition as a form of analgesia in chronic pain states including osteoarthritis and chronic low back pain [51].

### 3.1. Data from Preclinical Research

For the study of the pathogenesis of OA pain, several animal models have been developed. The most widely used models carry out surgical (transection of the medial meniscus and the anterior cruciate ligament) or chemical (intra-articular injection of monosodium iodoacetate, MIA) methods to induce pain.

Different molecular mechanisms involved in the pathogenesis of OA pain have been identified in these studies. Both surgical and MIA models develop inflammation in the synovium, subchondral changes, and cartilage degradation, and it has been demonstrated that the nociceptive state involves the activation of the NGF-TrkA axis in DRGs of the lumbar region [52].

Moreover, MIA model has shown an increase in CGRP expression on sensory nerve fibers in the DRG and synovium [53]. Regarding subchondral changes, the surgical model has shown increase in netrin-1 expression by osteoclasts, leading to sensory nerve axonal growth in subchondral bone [54]. On the other hand, locally in osteoarthritic joints, release of cytokines, chemokines, and inflammatory factors, including TNF, IL-1, IL-6, IL-17, NGF, and prostaglandin E2, can lead to exaggerated pain perception [54]. Even so, more research is needed to explain the biological effects of osteoarthritis damage.

The evaluation of pain in these OA animal models includes the most common behavioral assays: the von Frey test to assess mechanical allodynia, the weight bearing test to measure the distribution of the average weight load applied to each hind paw, the hot plate test that assesses thermal hyperalgesia, the rotarod test that evaluates changes in motor skills, and the evaluation of spontaneous pain by the measurement of ambulation and exploratory behavior [55].

In preclinical studies of OA, the therapeutic strategies to control pain are mainly based in the blockade of the NGF signaling that can be achieved using anti-NGF antibodies (see Table 2). Thus, some studies that used a surgical model of OA [56,57,58,59] or a MIA model in rats or mice [60,61,62,63,64] have reported the reversal effects or a protective role of the NGF blockade on pain behavior (mechanical allodynia, weight bearing, and gait deficiency) [56,60,61,63], on deficits in burrowing behavior [62], and on changes in joint structure [61,65] with treatments with anti-NGF antibodies (TrkAd5 [56], GZ389988 [60], AS2886401-00 [61], anti-NGF-2.5S [57], AR786 [66], muMab 911 [63], tanezumab [58], mAb911 [59], L148 M [64]). Most of these studies reported improvement of weight bearing [56,58,60,61,63,66] and gait deficiency [58], mechanical allodynia [57,63,66], and deficits in motor skills [59,62,64], even though the long-lasting analgesic effect on pain was not complemented by the suppression of knee edema and lesion score [61] or cartilage damage [58]. Moreover, the analgesic effect of the anti-NGF antibody NV-01 has been described in dogs with degenerative joint disease, who gained mobility and had decreased pain after treatment [67] (Table 2).

### 3.2. Data from Clinical Research

Several clinical trials for OA pain have been conducted in recent years with mAbs that neutralize NGF; tanezumab, fulranumab, and fasinumab appear particularly promising. They have demonstrated efficacy in phase II and III clinical trials in individuals with painful osteoarthritis and also low back pain (see Table 3). The endpoints taken into account in these studies were the Western Ontario and McMaster Universities Osteoarthritis Index (WOMAC) pain, Physical function, and Patient’s Global Assessment of OA (PGA-OA).

The most studied anti-NGF mAb in patients with OA has been tanezumab. Numerous clinical trials conducted over the last 20 years confirm its efficacy compared to placebo. Tanezumab relieves pain at week 4 after initiation of treatment, and this effect persists until week 16 or 24 [49,68,69,70,71,72,73,74,75]; moreover, it also improves the function and physician’s global assessment for both knee and hip OA. Some studies have compared the efficacy of tanezumab vs. NSAIDs, such as naproxen, celecoxib and diclofenac [69,76,77,78], or vs. opiates, such as oxycodone [79]. Meta-analyses performed on data from anti-NGF antibody clinical trials have shown that these agents have a significant but modest effect and are superior to placebo for the main study endpoints but have a variable effect in terms of superiority compared with NSAID treatments. Moreover, these meta-analyses reaffirmed the safety findings of the individual studies: anti-NGF antibodies increased peripheral neuropathy and sensitive adverse events (paresthesia, hypoesthesia), but there were no significant differences in serious adverse events compared with either placebo or NSAIDs [51].

Early clinical trials of tanezumab were aimed at studying the clinical efficacy of intravenous administration [68,69,76,77,79,80,81]; in contrast, more recent and ongoing trials study the efficacy of subcutaneous administration, and results seem to indicate that 5 mg is the minimum dose for achieve a valuable clinical reduction in pain and an improvement in quality of life [49,70,78,82,83].

In sum, anti-NGF antibodies are a promising therapeutic option in the treatment of OA, as they improve pain and function in patients; however, despite these benefits, meta-analyses of clinical trial data found that these agents increased the risk of neurological adverse effects (paresthesia, hypoesthesia, and peripheral neuropathy), being greater in patients treated with tanezumab than in patients treated with placebo and NSAIDs. Moreover, rapidly progressive osteoarthritis (RPOA) and fractures have been reported in some patients with hip/knee OA [51,84]; tanezumab-treated patients showed more joint safety events and total joint replacements [83]. RPOA was associated with higher doses of anti-NGF antibodies used alone or with NSAIDs, although the underlying molecular mechanism is unknown yet. The future research should be directed towards further study of the safety of these Abs in patients, and it is important to set up new clinical studies focused on the identification of risk factors of patients with OA who manifest RPOA.

## 4. mAbs in Rheumatoid Arthritis

Rheumatoid arthritis (RA) is a chronic autoimmune disease affecting almost 1% of the world’s population. It is characterized by systemic inflammation and persistent synovitis that affects the joints, mainly by a progressive articular disability. RA causes pain, stiffness, and swelling joints, leading to loss of physical function, work disability, and decreased quality of life of patients [85]. Its pathogenesis is still unclear, but it is known that pro-inflammatory cytokines such as IL-6 and TNF are involved in joint inflammation and extra-articular manifestations [86,87,88,89,90,91].

Pain in RA may be due to different etiologies, ranging from peripheral inflammation to dysregulation on the CNS processing. A large number of patients have symptoms consistent with neuropathic pain (allodynia and hyperalgesia) [92]. Therapeutic options to manage pain are focused on treating inflammation, and include NSAIDs, corticosteroids, weak opioids, and disease-modifying antirheumatic drugs (DMARDs) such as methotrexate. In addition, there are newer drugs, including mAb as TNF inhibitors and anti-IL-6 [90].

### 4.1. Data from Preclinical Research

An animal model that mimics human RA has not yet been achieved; despite this, two types of animal models that provide important observations about the pathogenesis of pain in RA have been developed: RA induced by active immunization and RA induced by transfer. The former type is the most commonly used and includes the collagen-induced arthritis (CIA) model, which is induced by the intradermal injection of collagen type II emulsified with Complete Freund’s Adjuvant (CFA) into the dorsal skin or just CFA injection in the tail; another model is the antigen-induced arthritis (AIA) developed by the injection of a mixture of methylated bovine serum albumin (mBSA) with CFA in the tail or knee joint of mice or rats [55]. On the other hand, the RA transfer model is induced by the injection of pathogenic autoantibodies or serum. In both models, joint inflammation and cartilage destruction occur, and inflammatory markers are increased. In addition, there are a variety of knockout or transgenic mice such as IL-1RA-KO, IL-6R knock-in, and TNF transgenic mice that are used to test the efficacy of new therapies [55].

The assessment of pain behavior in RA models is similar to that explained for OA models, the von Frey test to measure mechanical allodynia, and the hot plate test for thermal hypersensitivity. However, since asymmetric polyarthritis develops in RA models, it is not suitable to use the weight bearing test, although the catwalk test can be used. In both the CIA and AIA models, mechanical allodynia and thermal hypersensitivity are observed at the beginning of arthritis, but in the late phase, mechanical hypersensitivity persists, and thermal hyperalgesia disappears. In these RA models, functional impairment depends on joint destruction rather than inflammation [55].

In the treatment of pain that develops in animal models of RA, the effects of several mAbs against different targets have been studied (see Table 2). Thus, using the CIA mouse model, treatment with two antibodies directed against IL-6: hyaluronate gold nanoparticle/tocilizumab complex and tocilizumab, induced a decrease in the inflammatory cell infiltration and destruction of cartilage and bone [93]. Moreover, the monoclonal antibody NI-0101 directed to block TLR4 was capable of inhibiting the release of cytokines induced by LPS in a CIA mouse model and to improve the disease progression [94]. Furthermore, the selective inhibition of TrkA with the mAb AR786 would reduce pain behaviors and synovial inflammation in the CIA model [95]. Regarding TNF, in the AIA rat model, treatment with the anti-TNF antibody infliximab reduced swelling and mechanical hyperalgesia in the inflamed knee while not reducing thermal hyperalgesia [96]. Moreover, in a transgenic mouse model of RA that overexpresses TNF, infliximab also improved arthritic symptoms and neurological function [97], and the use of another anti-TNF mAb, adalimumab, reduced signs of inflammation and edema of the affected joints, in addition to decreasing the morphological signs of the disease and of the expression of TNF in a rat model of RA induced by CFA [98].

On the other hand, the mAb directed against the urokinase-type plasminogen activator (uPA) mU1 neutralized the progression of the disease both in the CIA and AIA models in mice, and the injection of mAbs against adiponectin (KH7-33 and KH4-8) can inhibit arthritic symptoms (arthritis index, squeaking index, and the volume of the paw) in the CIA mouse model; a slight decrease in the levels of TNF and IL-6 was also observed, but without a decrease in the expression of adiponectin. These changes were correlated with a decrease of the area of inflammation and of the degradation of the joint cavity [99]. Finally, using the AIA mouse model, researchers have reported that the acute local blockade with an anti-FcγRI mAb (anti-CD64) reduced arthritis pain, although it did not modify joint inflammation or the infiltration of immune cells into inflamed synovium [100] (Table 2).

### 4.2. Data from Clinical Research

There are numerous clinical trials of RA patients testing the efficacy of TNF inhibitors (infliximab, adalimumab, golimumab, and certolizumab) and anti-IL-6 (tocilizumab) [90], alone or in combination with other drugs such as methotrexate [101,102]; the results show some overall improvement of the disease, as these drugs provide rapid symptom relief by decreasing inflammation and improving other complications associated with RA (fatigue, sleep, and mood disorders), but there are few studies that specifically focus on evaluating pain (see Table 3).

Clinical trials in patient non-responders to methotrexate showed that tocilizumab significantly improved pain, as measured by a visual analog scale [103,104,105], and improved fatigue [106]. Data from meta-analysis confirm that IL-6 can be targeted to relieve inflammatory pain, and anti-IL-6 or anti-IL-6 receptor agents seem to alleviate allodynia and hyperalgesia [86].

TNF inhibitors prevent the recruitment of the cells that cause inflammation, bring rapid symptom relief, decrease pain, improve function, and ameliorate progressive joint damage in RA, but they are recommended when other drugs have failed; unfortunately, these drugs are very expensive, and their efficacy in different stages of RA is still under study. They are usually administered concomitantly with methotrexate and are contraindicated in patients with congestive heart failure of demyelinating diseases; these agents are also immunosuppressive and can produce reactivation of latent infections [90,107].

Rituximab is useful in cases of RA where TNF inhibitors are not effective; it depletes the B cells responsible for inflammation and the production of abnormal antibodies and improves the complications of RA (vasculitis and cryoglobulinemia) [108].

## 5. mAbs in Migraine

Migraine is one of the most common painful pathologies that causes disability in people who suffer from it. Patients suffering from this disease are usually treated with analgesics, but some of them, due to the frequency, intensity, and impact on their quality of life, need preventive treatment to reduce the occurrence of acute episodes and analgesics to relieve their pain [109]. CGRP has been shown to be involved in both pain transmission and inflammation, and is considered to have a major role in migraine pathogenesis [110,111]; it has a broad spectrum of activity in migraine pain transmission, producing vasodilatation, edema, and mast cell degranulation at the peripheral level (trigeminal nerve endings), contributing to neurogenic inflammation. In the trigeminal ganglion, CGRP activates glial cells with the consequent release of proinflammatory cytokines and sensitization of sensory nerves; moreover, from the ganglion, there is projection and transmission of pain to the thalamus, which contributes to the development of migraine and photophobia [17,112,113].

### 5.1. Data from Preclinical Research

Through the years, numerous animal models have been developed to study migraine, showing the involvement of CGRP in peripheral (trigeminal nerve) and central (meninges) sensitization. Thus, animal models of migraine induced by the administration of CGRP, both by peripheral and central routes (intravenous, intraperitoneal, subcutaneous, intradermal, dural, epidural, intracerebroventricular, and intrathecal) has been developed [114,115]. When this migraine animal model was used, treatment of mice with the anti-CGRP mAb ALD405 was able to attenuate the aversion to light and avoid the reduced motility [116], and, furthermore, the administration of ALD405 completely blocked the spontaneous pain behavior and grimaces while also preventing piloerection, diarrhea, and cutaneous vasodilation [117] (Table 2).

### 5.2. Data from Clinical Research

mAbs acting on the CGRP or its receptor have been studied in numerous phase II or III clinical trials and have shown significant efficacy for either the acute or preventive treatment of migraine [110,118]. The anti-CGRP mAbs developed and studied in patients with episodic and chronic migraine to date are erenumab, eptinezumab, fremanezumab, and galcanezumab [109,110], all of them approved by the U.S. Food and Drug Administration (FDA) and the EMA to treat migraine [110] (see Table 3). Most of these agents target the CGRP peptide, except erenumab, which targets the CGRP receptor. All these treatments have led to significant reductions from baseline in either episodic [119,120,121,122] and/or chronic [123,124,125,126] monthly migraine or headache days vs. placebo. A variety of secondary endpoints were also achieved in the clinic trials, such as reduction in acute medication and several impact and disability measures [110]. All studies suggest that the anti-CGRP mAbs are well tolerated, demonstrating an acceptable safety profile [109,110]. Eptinezumab is the only one of this group that can be injected intravenously [127].

Recommendations indicate that these treatments should be offered to patients with migraine when at least two of the available medical treatments have failed or when the preventive treatments cannot be used because of comorbidities, side effects, or poor compliance [109,110].

## 6. mAbs in Chronic Low Back Pain

Advances in preclinical and clinical research have led to the development of biological agents targeting specific cytokines in the potentiation and transmission of pain in chronic low back pain (CLBP) where inflammatory processes occur; these targets are mainly TNF and NGF [128] (see Table 3).

TNF is an important inflammatory mediator that irritates and damages nerve roots. In the first decade of the 2000s, there were several clinical studies that investigated the effectiveness of anti-TNF therapy (infliximab, etanercept, and adalimumab) in CLBP with or without radiculopathy, or sciatica [17], but randomized controlled trials and a meta-analysis failed to demonstrate the superiority of anti-TNF vs. placebo [128,129,130]. Although there is insufficient evidence to recommend this therapy, the fact that in some trials there has been a significant reduction in pain intensity makes it interesting for further study.

Conversely, mAbs targeting NGF have shown clinical efficacy in this type of pain [51], especially tanezumab, which induces pain relief and functional improvement [131,132,133,134]. In some phase III clinical trials, tanezumab provided significantly greater improvement in pain, functional capacity, and global scores vs. placebo and naproxen in patients with CLBP [132,133], but it was not superior in comparison with tramadol; it was also associated with a low rate of joint safety events, some requiring joint replacement.

Considering the results of all the clinical studies that have evaluated these mAbs in patients with low back pain, the results are not satisfactory; the systematic reviews and meta-analyses demonstrate the low quality of the trials and their high heterogeneity in terms of sample size, doses, routes of administration, duration, and follow-up of treatment [128]. It is therefore mandatory to continue studying the use of these agents in well-designed trials. Low back pain is very disabling in younger patients, making this population group an important target for investigating the efficacy of mAbs, especially when other therapeutic treatments fail.

## 7. mAbs in Neuropathic Pain

Neuropathic pain constitutes the 15–25% of chronic pain cases. The International Association for the Study of Pain (IASP) has defined it as “pain caused by a lesion or disease of the somatosensory nervous system” [135]. Thus, neuropathic pain includes multiple subtypes, and while numerous preclinical articles have been published, there is much less clinical evidence.

### 7.1. Data from Preclinical Research

Painful diabetic neuropathy (PDN) and chemotherapy-induced peripheral neuropathy (CIPN) are two types of painful pathologies for which animal models of pain have been developed.

Diabetes mellitus is a disease that usually presents PDN as a disabling chronic complication for patients. An animal model of diabetes type 2 that is commonly used in the study of effective treatments for the symptoms that patients suffer is the homozygous diabetic mouse (Lepr^db^/Lepr^db^, also named db/db) [37,136,137]. This rodent model, among other symptoms, induces pain sensibility that involves a pathologically altered activity of the intraepidermal nerve fibers (IENFs) of primary afferent Aδ- and C-fibers that innervate the skin [136], along with neuroinflammation and astrocytic activation in the spinal dorsal horn. Thus, different immunological factors are implicated in the development and maintenance of PND, and among them, the role of the protein HMGB1 has been highlighted, for which expression significantly increases in db/db mice, mainly as a consequence of the oxidative stress and other pathological pathways induced or activated in this disease by the hyperglycemic state; this correlates with mechanical allodynia in the hind paws, that is present when mice are 2 months old and lasts for at least 3 months.

In this animal model of diabetes, the injection of polyclonal neutralizing Ab against HMGB1 decreased in a dose-dependent manner the astrocytic activation, downregulated the expression of inflammatory mediators, and reduced the mechanical allodynia [37]. Further, it has been demonstrated that treatment with an anti-NGF mAb (clone AS21) attenuated the mechanical allodynia by a peripheral mechanism that involves the decrease of the density of TrKA-positive cells that also are substance P- and CGRP-positive [136] (Table 4).

CIPN is a limiting side effect that appears in a high percentage of patients and is caused by some anticancer drugs that are commonly used in chemotherapeutic treatments, such as platinum compounds (oxaliplatin, cisplatin), vinca alkaloids (vincristine), and taxanes (paclitaxel). The presence of CIPN sometimes results in a discontinuity of the treatment and includes signs of peripheral neuropathy that evokes sensorial and nociceptive alterations (paresthesias and dysesthesias, hypersensitivity to thermal and/or mechanical stimuli, numbness and neuropathic spontaneous pain) mainly in hands and feet [138,139]; for many patients, the medication commonly used to treat these signs is not effective.

The underlying mechanism of the neurotoxicity induced by these agents involves several neuropathological mechanisms including neuroinflammation [135]. The prevention of the proinflammatory cytokine release evoked by the activation of macrophages and satellite cells in the DRG could constitute a therapeutic target.

Among the animal models developed for the study of CIPN, one of the most commonly used is the repeated intraperitoneal administration of paclitaxel [140], which after a few days post-treatment time-dependently induces signs of motor and sensory peripheral neuropathies in the animal paws, associated with impaired motor coordination and behavioral dysfunction (mechanical allodynia and heat hyper- or hypoalgesia) by the induction of inflammation in DRG tissues and damage (axon demyelination) in both sensory and motor nerve fibers and loss of hind paw skin IENFs. These neuropathic signs are fully established on day 14 after the start of treatment with paclitaxel and last at least for 28 days [30].

In this regard, a significant transcriptional upregulation of MMP2 and MMP9 in DRG neurons has been described. The acute and repeated treatment with the MMP9 mAb that targets the human and mouse MMP-9 (MMP9 mAb, clone 6-6B) was able to reverse and prevent, respectively, the mechanical allodynia developed by mice; the oxidative stress caused by ROS production; and the neuroinflammation induced by the expression of the proinflammatory cytokines IL-6 and TNF, as well as the proalgesic inducible nitric oxide synthetase (NOS2). This mAb protected against the degeneration of IENFs associated with the CIPN [30] (Table 4).

The repeated administration of a neutralized monoclonal anti-IL-20 antibody (7E) and a mAb of the IL-20 receptor 1 (51D), once the neuropathy was developed, were able to ameliorate the nerve damage and consequently attenuate the mechanical allodynia, prevent the thermal hypoesthesia, and improve the defects in motor coordination. Thus, IL-20 constitutes another interesting target for the prevention and treatment of CIPN by the clinical use of neutralizing or receptor-blocking mAbs in patients that are receiving chemotherapy [34] (Table 4).

Other rodent models of peripheral neuropathic pain (PNP) have been used to study the effects of Abs. A classic model of PNP consists of the ligation or transection of a peripheral nerve, which after some time develops a neuropathic state with characteristic signs of hypersensitivity in the affected limbs, as occurs in the rodent neuropathic pain model of the partial sciatic nerve ligation (PSNL) that induces in the injured nerve an upregulation of pronociceptive molecules that support the maintenance of the neuropathic pain state. Thus, after 7 days post-injury, animals show a significant mechanical allodynia in the ipsilateral hind paw that lasts at least 21 days and, after 14 days of the PSNL, is associated with macrophage infiltration and Schwann cell proliferation and elevated mRNA expression of IL-1β, TNF, MMP-9, cyclooxygenase-2 (COX-2), and HMGB1 in the ipsilateral injured sciatic nerve. Using this model in mice, researchers found a decrease of the mechanical hypersensitivity by the repeated administration of an anti-HMGB1 rat monoclonal antibody (anti-HMGB1 mAb (#10-22, IgG2a subclass)) and also a delayed expression of MMP-9 and IL-1β in both macrophage and Schwann cells at this time (Table 4). These results point out a possible HMGB1-NF-kβ-MMP9 cascade mediating the maintenance of neuropathic hypersensitivity to pressure stimuli in the injured nerve, and the blockade of this cascade constitutes a key strategy for the amelioration of neuropathic pain [35].

Other studies also demonstrated the possibility of reducing mechanical allodynia and thermal hyperalgesia through suppression on accumulated neutrophils and macrophages of the upregulation of inflammatory chemokines and cytokines MIP-2 and CXCR2 in the injured sciatic nerve with the MIP-2-neutralizing antibody (anti-MIP-2) [141].

Furthermore, some studies have also shown the effects of blocking NGF effects with the administration of mAbs in a peripheral neuropathic pain model. For example, when the chronic constriction injury (CCI) model in mouse was used, an anti-NGF mouse mAb applied 2 weeks after surgery evoked a sustained reversal of the mechanical hypersensitivity, which remained 5 days after the end of the treatment [142]. Moreover, anti-NGF treatment was demonstrated to be effective in suppressing the mechanical hyperalgesia and the hyper-responsiveness of wide dynamic range neurons in the spinal cord [143].

### 7.2. Data from Clinical Research

In contrast to preclinical research, clinical evidence for the usefulness of mAbs in neuropathic pain is still limited. For diabetic peripheral neuropathy (DPN) and postherpetic neuralgia (PHN), two randomized controlled trials (phase II) were conducted to study the efficacy and safety of the anti-NGF mAb, tanezumab [144] (Table 3). This drug significantly reduced the pain in patients suffering of DPN vs. placebo; conversely, it did not improve the pain in PHN. It is unclear why these patient populations had different responses to tanezumab. Mechanisms underlying neuropathic pain are diverse and may include variations in the degree, type, and severity of neuropathy. It is possible that mechanisms and pathophysiology of DPN may differ considerably from those of PHN, particularly with regards to the involvement of NGF. In relation to safety, there were no increase in adverse events vs. placebo [145].

Furthermore, there are some clinical studies that evaluated the pharmacokinetics or efficacy of mAb in oncological conditions in which neuropathic pain is a symptom and is considered a secondary outcome measure, as for example, rituximab treatment in patients with lymphoma [146,147].

## 8. Other Clinical Evidence of Use of mAbs in Chronic Pain: Endometriosis and Fibromyalgia

- Endometriosis: deep endometriosis-associated pain is believed to be caused by inflammation. Endometriosis is associated with an inflammatory response in the pelvis, which is mediated by several cytokines including TNF. It has therefore been suggested that the anti-TNF mAb infliximab might relieve pain in affected women. Thus, a phase II clinical trial to study infliximab effect was conducted in women with endometriosis [148], and results indicated that it did not appear to modify pain [149].
- Fibromyalgia: currently, a phase II clinical trial is ongoing to estimate the effect of fremanezumab administered subcutaneously in reducing pain in adult patients with fibromyalgia. Other measures of efficacy will also be studied, such as quality of life, sleep, fatigue, health improvement, physical functioning, and mood, as well as the safety and tolerability of this drug [150]. This study is currently in the recruitment phase.

## 9. Future Applications of mAbs in Preclinical Development: Cancer Pain and Pain in Bone Fracture

- Bone cancer pain: among other adverse consequences, the tumor formation in bones provokes in many patients a severe pain state (including spontaneous and evoked) that impairs quality of life. This pain state includes several aspects such as nociceptive, inflammatory, and neuropathic effects.

A model of cancer-induced bone pain could be developed in rodents via the injection of cancer cells in the medulla of the tibia [38,151,152], and after several days of the tumor inoculation, it induces signs that correlate with clinical symptoms in patients [38]. Moreover, the tumor induces damage to peripheral nociceptors (TrkA-positive Aδ- and C-nociceptive fibers) that innervate the bone and become sensitized through the release of several neurotransmitters, which is considered the major contributor of pain [153].

Additionally, NGF induces sprouting and hyper-innervation of bones [43]; thus, an-ti-NGF Abs used to treat cancer pain were largely described to be highly effective in decreasing peripheral nerve sprouting, neuroma formation, and behavioral nociceptive and spinal cord signs in mice [43,154] (see Table 4).

On the other hand, it has been found that from day 9 of the cancer cell inoculation in-to the tibia medulla, there was an increased expression of both the protein HMGB1 and the IL-1β in the spinal dorsal horn that correlated with the loss and destruction of the bone and with a progressive allodynia to pressure stimulation in the ipsilateral inoculated hind paw. The administration of a polyclonal neutralizing Ab against HMGB1 (anti-HMGB1) significantly reversed the mechanical allodynia, and this anti-allodynic effect lasted for 1 day. Thus, the blockade of spinal HMGB1 could attenuate bone cancer pain and downregulate the expression of IL-1β in a dose-dependent manner [38].

Furthermore, when the peripheral effects of the treatment with the anti-NGF mono-clonal antibody mAb911 were assessed in a mouse model of bone cancer pain in which symptoms that reproduce clinical symptoms correlated with the tumor growth and bone remodeling develop [151], this antibody induced a decrease in spontaneous pain behavior [43]. The antinociceptive effectiveness of mAb911 in cancer-induced bone pain was also demonstrated in another study [154].

Clinical studies in cancer pain are very scarce and more are needed to know whether these drugs could have clinical utility.

- Pain in bone fracture: the mouse model of painful bone fracture induces a marked reduction in the physical activity of the animal; using this model, in which mice exhibited femur fracture pain, it has been found that the administration of the anti-NGF antibody mAb911 induced an improvement in mice physical activity by blocking the sensitization of nociceptors that innervate the bone [155] (Table 4).

As a summary, Table 2, Table 3 and Table 4 show the different chronic pain conditions in which the usefulness of mAbs has been studied in different preclinical and clinical studies.

**Table 2:** Monoclonal antibodies evaluated in preclinical studies for chronic pain treatment.

Animal Model	Target	mAb	Method	Species	Pain Assessed	Effect	Reference
Osteoarthritis	NGF	GZ389988	Chemical MIA	Lewis rats	Weigh bearing	Reduction of weight bearing imbalance	[60]
		Tanezumab	Surgical MMT	Lewis rats	Weigh bearing	Gait deficiency prevention	[58]
		AS2886401-00	Chemical MIA	Sprague-Dawley rats	Gait analysis	Improvement in gait imbalance, no effect on knee lesion score	[61]
		AR786	Surgical MNX, Chemical MIA	Sprague-Dawley rats	Mechanical sensitivity, weight-bearing	Reduction of mechanical allodynia, reversal of weight-bearing asymmetry	[66]
		muMab 911	Chemical MIA	Sprague-Dawley rats	Mechanical sensitivity, weight-bearing	Reduction of mechanical allodynia, reversal of weight-bearing asymmetry	[66]
		WO 2004/058184 A2	Chemical MIA	Wistar Han rats	Spontaneous locomotor activity	Reverse deficits in burrowing	[62]
		NV-01	Spontaneous degenerative joint disease	Dogs	Spontaneous activity, pain	Gain mobility, improve pain severity	[67]
		TrkAd5	Surgical DMM	C57BL/6 mice	Weight-bearing	Improvement of weight bearing deficit	[56]
		Anti-NGF-2.5S	Surgical DMM	PKCδ null mice	Mechanical sensitivity	Reduce mechanical allodynia	[57]
		mAb911	Surgical arthrotomy	C3H/HeJ mice	Spontaneous locomotor activity	Increase of horizontal activity, vertical rearing, and horizontal velocity	[59]
		L148 M	Chemical MIA	C57BR/J mice	Gait analysis	Improvement of duty cycle, swing speed, and print area	[64]
Rheumatoid arthritis	IL-6	HA-AuNP/TCZ, TCZ	CIA	DBA/1j mice	Clinical scores	Improvement in the degree of swelling	[93]
	TLR4	TLR4 blocker NI-0101	CIA	Mice		Inhibition of LPS-induced cytokine release	[94]
	NGF	AR786	CIA, Carragenan	Sprague-Dawley rats	Mechanical sensitivity, weight-bearing, joint swelling	Reduce mechanical allodynia and weight-bearing asymmetry, inhibit partially knee swelling	[95]
	TNF	Infliximab	AIA	Lewis rats	Mechanical and thermal sensitivity, joint swelling	Reduce mechanical hyperalgesia and swelling in inflamed joint	[96]
			TNF transgenic	TNF transgenic mice	Clinical scoring	Averted the increase of symptom severity	[97]
		Adalimumab Humira	CFA	Wistar rats	Morphological examination of the metatarsophalangeal joints	Decrease of inflammation signal	[98]
	uPA	Ab anti-uPA mU1	CIA, AIA	DBA/1 mice	Clinical scores	Inhibition of disease progression	[156]
	Adiponectin	mAbs KH7–33 and KH4–8	CIA	DBA/1 J mice	Arthritis and squeaking index, paw volume	Inhibit arthritic symptoms	[99]
	FcγRI	anti-CD64	AIA	C57BL/6 mice	Mechanical and thermal sensitivity, joint diameter	Reductions in mechanical and thermal hyperalgesia	[100]
Migraine	CGRP	ALD405	CGRP-induced	CD1 mice	Light aversion and motility assessment	Aversion to light attenuated, reduced motility avoided	[116]
					Mouse grimace scale	Blockade of facial signs of discomfort	[117]

AIA: antigen-induced arthritis; CFA: complete Freund’s adjuvant; CGRP: calcitonin gene-related peptide; CIA: collagen-induced arthritis; DMM: destabilization of the medial meniscus; FcγRI: fragment crystallizable gamma receptor 1; HA-AuNP/TCZ: hyaluronate gold nanoparticle/tocilizumab complex; IL-6: interleukin-6; MIA: monosodium iodoacetate; MMT: medial meniscal tear; MNX: meniscal transection; NGF: nerve growth factor; TCZ: tocilizumab; TLR4: toll-like receptor 4; TNF: tumor necrosis factor; TrkAd5: domain 5 of the tropomyosin receptor kinase A; uPA: urokinase-type plasminogen activator.

**Table 3:** Chronic pain conditions in which the efficacy of mAbs has been clinically studied.

Type of Pain	Target	mAb	Main Findings	Reference
Osteoarthritis (hip or knee)	NGF	Tanezumab	Decreases joint pain and improves physical function. Neurosensory and neuromuscular adverse events. Risk of RPOA at high doses.	[49,68,69,70,71,72,76,77,78,79,80,81,82,83,157]
		Fulranumab	Greater pain relief relative to oxycodone. Arthralgias and risk of RPOA associated to treatment.	[73,74]
		Fasinumab	Decreases joint pain and improves physical function. Arthropathies associated to treatment.	[75]
Chronic low back pain	NGF	Tanezumab	Improvement in pain, function, and global scores vs. placebo and naproxen. Arthralgias and risk of neurological adverse events.	[131,132,133,134]
	TNF	Infliximab	No results yet.	[158]
Migraine	CGRP	Erenumab	Migraine prevention in patients with chronic or episodic migraine. Reduction in monthly migraine days. Low adverse event burden.	[109,119,120,123,159,160,161,162,163]
		Eptinezumab	Reduction in monthly migraine days in chronic migraine. Nasopharyngitis adverse event.	[124,127]
		Fremanezumab	Reduction of headache severity and duration in patients with chronic or episodic migraine. Decrease in consumption of acute migraine medications.	[125,164,165,166,167,168]
		Galcanezumab	Reduction in monthly migraine days in chronic or episodic migraines. Improvement in function. Mild pain and erythema in injection site.	[169,170]
Rheumatoid arthritis	IL-6	Sarilumab	Improvement in pain and fatigue.	[86,87,171,172]
		Tocilizumab	Improvement in signs and symptoms. Greater inhibition of joint damage and improvement in physical function with tocilizumab plus MTX vs. MTX alone. Infections adverse events.	[103,104,173]
	TNF	Adalimumab	Improvement in pain and physical function. Risk of serious infections.	[174,175]
		Golimumab	Improvement in signs and symptoms with golimumab plus MTX vs. MTX alone. Infections adverse events.	[176,177]
		Certolizumab	Improvement in signs and symptoms, pain, fatigue, and health-related quality of life. Increased chance of remission of RA and reduced joint damage. Respiratory tract infections.	[178,179]
		Infliximab	Pain relief and inhibition of cartilage destruction. Decrease in the expression of inflammatory cytokines in the synovial fluid and cartilage.	[180,181]
	CD20 in B cells	Rituximab	Improvement in physical function and health-related quality of life with rituximab plus MTX vs. MTX alone. Risk of serious infections.	[108,182]
Neuropathic pain (DPN, PHN)	NGF	Tanezumab	Pain reduction in DPN. Pain reduction in PHN only at the highest dose.	[144,145]

NGF: nerve growth factor; TNF: tumor necrosis factor; CGRP: calcitonin gene-related peptide; IL-6: interleukin 6; RPOA: rapidly progressive osteoarthritis; MTX: methotrexate; DPN: diabetic peripheral neuropathy; PHN: postherpetic neuralgia.

**Table 4:** Monoclonal antibodies evaluated in preclinical studies for neuropathic pain and other animal models of pain.

Animal Model	Target	mAb	Method	Species	Pain Assessed	Effect	Reference
Neuropathic pain	MMP9	MMP9 mAb clone 6-6B	Chemical paclitaxel	CD1 mice	Mechanical and thermal sensitivity	Reduction and prevention of mechanical allodynia	[30]
	IL-20	anti-IL-20 mAb (7E), anti-IL-20R1 mAb (51D)	Chemical paclitaxel	C57BL/6J mice	Mechanical and thermal sensitivity, motor impairment	Attenuation of mechanical allodynia, heat hypoesthesia and defects in motor coordination	[34]
	NGF	anti-NGF mAb (clone AS21)	Diabetes development	db/db mice	Mechanical sensitivity	Decrease of mechanical allodynia	[136]
	HMGB1	anti-HMGB1 mAb (#10-22, IgG2a subclass)	Partial sciatic nerve ligation	ddy mice	Mechanical sensitivity	Decrease of mechanical allodynia	[35]
Cancer pain	NGF	mAb911	Tibial tumor inoculation	C57BL/6 mice	Spontaneous pain behavior	Decrease in guarding and flinching behavior of the affected hind paw	[43]
Bone fracture pain	NGF	mAb911	Femur fracture	C57Bl/6J mice	Spontaneous locomotor activity	Higher horizontal distance travelled, increase of number of rearing episodes and average velocity	[155]

MMP9: matrix metalloproteinase 9; IL-20: interleukin-20; NGF: nerve growth factor; HMGB1: high-mobility group box-1.

## 10. Conclusions

Currently, although there are numerous therapeutic pharmacological options available to treat chronic pain, these options still present limitations due to lack of efficacy or undesirable adverse effects. The large number of mAbs available have facilitated their study in preclinical research for later clinical use in the treatment of several diseases. Preclinical studies are improving the understanding of the mechanisms by which mAb are useful in the treatment of chronic pain and are demonstrating their efficacy in various animal models of pain. In clinical practice, the painful conditions for which mAb are recommended are more limited, but there is increasing evidence of the usefulness of mAbs in musculoskeletal pain, such as knee and hip osteoarthritis and chronic low back pain, rheumatoid arthritis, or migraine. As well as relieving pain, these agents improve patients’ physical function and quality of life.

However, more work should be done in order to clarify the central antinociceptive effects of mAbs and to understand their role in central sensitization. An effort should be carried out to use central ways of administration and/or new delivery technologies to enable mAbs entry into CNS across the BBB from blood.

In summary, the analgesic efficacy of mAbs, accompanied by a reduction in adverse effects and a better adherence to treatment in comparison to conventional analgesic medication, indicate that this family of drugs could be in a future an interesting pharmacological option to treat chronic pain and ameliorate issues with patients’ quality of life.

## Figures and Tables

**Table 1:** Some pharmacological differences between mAbs and classical drugs.

Monoclonal Antibodies	Classic Drugs
Very high selectivity (few side effects)	Good selectivity (dose-related)
Parenteral administration	Multiple routes of administration
PK: elimination by either excretion or catabolism	PK: classic metabolism and excretion (liver, kidney, etc.)
PK and PD interactions almost excluded	PK and PD interactions
Prolonged half-life	Shorter half-life
Produced by genetic engineering	Chemical synthesis or natural purification
Do not cross BBB	Can cross BBB
Can produce immunogenicity	Poorly immunogenic

PK = pharmacokinetics; PD = pharmacodynamics; BBB = blood–brain barrier.

## References

[1] Buss N.A.P.S., Henderson S.J., McFarlane M., Shenton J.M., de Haan L. (2012). Monoclonal Antibody Therapeutics: History and Future. Curr. Opin. Pharmacol..

[2] Parray H.A., Shukla S., Samal S., Shrivastava T., Ahmed S., Sharma C., Kumar R. (2020). Hybridoma Technology a Versatile Method for Isolation of Monoclonal Antibodies, Its Applicability across Species, Limitations, Advancement and Future Perspectives. Int. Immunopharmacol..

[3] Kaunitz J.D. (2017). Development of Monoclonal Antibodies: The Dawn of MAb Rule. Dig. Dis. Sci..

[4] Parren P.W.H.I., Carter P.J., Plückthun A. (2017). Changes to International Nonproprietary Names for Antibody Therapeutics 2017 and beyond: Of Mice, Men and More. mAbs.

[5] Bayer V. (2019). An Overview of Monoclonal Antibodies. Semin. Oncol. Nurs..

[6] Jones T.D., Carter P.J., Plückthun A., Vásquez M., Holgate R.G.E., Hötzel I., Popplewell A.G., Parren P.W.H.I., Enzelberger M., Rademaker H.J. (2016). The INNs and Outs of Antibody Nonproprietary Names. mAbs.

[7] Shepard H.M., Phillips G.L., Thanos C.D., Feldmann M. (2017). Developments in Therapy with Monoclonal Antibodies and Related Proteins. Clin. Med..

[8] García Merino A. (2011). Monoclonal Antibodies. Basic Features. Neurolia.

[9] Lu R.-M., Hwang Y.-C., Liu I.-J., Lee C.-C., Tsai H.-Z., Li H.-J., Wu H.-C. (2020). Development of Therapeutic Antibodies for the Treatment of Diseases. J. Biomed. Sci..

[10] Similar Biological Medicinal Products. https://www.ema.europa.eu/en/similar-biological-medicinal-products.

[11] Declerck P., Danesi R., Petersel D., Jacobs I. (2017). The Language of Biosimilars: Clarification, Definitions, and Regulatory Aspects. Drugs.

[12] Castelli M.S., McGonigle P., Hornby P.J. (2019). The Pharmacology and Therapeutic Applications of Monoclonal Antibodies. Pharmacol. Res. Perspect..

[13] Pandey M., Mahadevan D. (2014). Monoclonal Antibodies as Therapeutics in Human Malignancies. Future Oncol..

[14] Balakrishnan P.B., Sweeney E.E., Ramanujam A.S., Fernandes R. (2020). Photothermal Therapies to Improve Immune Checkpoint Blockade for Cancer. Int. J. Hyperth..

[15] Charmsaz S., Scott A.M., Boyd A.W. (2017). Targeted Therapies in Hematological Malignancies Using Therapeutic Monoclonal Antibodies against Eph Family Receptors. Exp. Hematol..

[16] Raja S.N., Carr D.B., Cohen M., Finnerup N.B., Flor H., Gibson S., Keefe F.J., Mogil J.S., Ringkamp M., Sluka K.A. (2020). The Revised International Association for the Study of Pain Definition of Pain: Concepts, Challenges, and Compromises. Pain.

[17] Yeh J.-F., Akinci A., Al Shaker M., Chang M.H., Danilov A., Guillen R., Johnson K.W., Kim Y.-C., El-Shafei A.A., Skljarevski V. (2017). Monoclonal Antibodies for Chronic Pain: A Practical Review of Mechanisms and Clinical Applications. Mol. Pain.

[18] Heinricher M.M. (2016). Pain Modulation and the Transition from Acute to Chronic Pain. Adv. Exp. Med. Biol..

[19] Ji R.-R., Nackley A., Huh Y., Terrando N., Maixner W. (2018). Neuroinflammation and Central Sensitization in Chronic and Widespread Pain. Anesthesiology.

[20] Haight E.S., Forman T.E., Cordonnier S.A., James M.L., Tawfik V.L. (2019). Microglial Modulation as a Target for Chronic Pain: From the Bench to the Bedside and Back. Anesth. Analg..

[21] Szok D., Tajti J., Nyári A., Vécsei L. (2019). Therapeutic Approaches for Peripheral and Central Neuropathic Pain. Behav. Neurol..

[22] Matsuda M., Huh Y., Ji R.-R. (2019). Roles of Inflammation, Neurogenic Inflammation, and Neuroinflammation in Pain. J. Anesth..

[23] Bannwarth B., Kostine M. (2015). Biologics in the Treatment of Chronic Pain: A New Era of Therapy?. Clin. Pharmacol. Ther..

[24] Mai L., Zhu X., Huang F., He H., Fan W. (2020). P38 Mitogen-Activated Protein Kinase and Pain. Life Sci..

[25] Edelmayer R.M., Brederson J.-D., Jarvis M.F., Bitner R.S. (2014). Biochemical and Pharmacological Assessment of MAP-Kinase Signaling along Pain Pathways in Experimental Rodent Models: A Potential Tool for the Discovery of Novel Antinociceptive Therapeutics. Biochem. Pharmacol..

[26] McKelvey L., Shorten G.D., O’Keeffe G.W. (2013). Nerve Growth Factor-Mediated Regulation of Pain Signalling and Proposed New Intervention Strategies in Clinical Pain Management. J. Neurochem..

[27] Kim S.H., Nam J.S., Choi D.K., Koh W.W., Suh J.H., Song J.G., Shin J.W., Leem J.G. (2011). Tumor Necrosis Factor-Alpha and Apoptosis Following Spinal Nerve Ligation Injury in Rats. Korean J. Pain.

[28] Kim Y., Remacle A.G., Chernov A.V., Liu H., Shubayev I., Lai C., Dolkas J., Shiryaev S.A., Golubkov V.S., Mizisin A.P. (2012). The MMP-9/TIMP-1 Axis Controls the Status of Differentiation and Function of Myelin-Forming Schwann Cells in Nerve Regeneration. PLoS ONE.

[29] Kawasaki Y., Xu Z.-Z., Wang X., Park J.Y., Zhuang Z.-Y., Tan P.-H., Gao Y.-J., Roy K., Corfas G., Lo E.H. (2008). Distinct Roles of Matrix Metalloproteases in the Early- and Late-Phase Development of Neuropathic Pain. Nat. Med..

[30] Tonello R., Lee S.H., Berta T. (2019). Monoclonal Antibody Targeting the Matrix Metalloproteinase 9 Prevents and Reverses Paclitaxel-Induced Peripheral Neuropathy in Mice. J. Pain.

[31] Bigal M.E., Walter S., Rapoport A.M. (2013). Calcitonin Gene-Related Peptide (CGRP) and Migraine Current Understanding and State of Development. Headache.

[32] Sebba A. (2021). Pain: A Review of Interleukin-6 and Its Roles in the Pain of Rheumatoid Arthritis. Open Access Rheumatol. Res. Rev..

[33] Kang S., Tanaka T., Kishimoto T. (2015). Therapeutic Uses of Anti-Interleukin-6 Receptor Antibody. Int. Immunol..

[34] Chen L.-H., Yeh Y.-M., Chen Y.-F., Hsu Y.-H., Wang H.-H., Lin P.-C., Chang L.-Y., Lin C.-C.K., Chang M.-S., Shen M.-R. (2020). Targeting Interleukin-20 Alleviates Paclitaxel-Induced Peripheral Neuropathy. PAIN.

[35] Zhang F.F., Morioka N., Harano S., Nakamura Y., Liu K., Nishibori M., Hisaoka-Nakashima K., Nakata Y. (2016). Perineural Expression of High-Mobility Group Box-1 Contributes to Long-Lasting Mechanical Hypersensitivity via Matrix Metalloprotease-9 up-Regulation in Mice with Painful Peripheral Neuropathy. J. Neurochem..

[36] Maeda T., Ozaki M., Kobayashi Y., Kiguchi N., Kishioka S. (2013). HMGB1 as a Potential Therapeutic Target for Neuropathic Pain. J. Pharmacol. Sci..

[37] Ren P.-C., Zhang Y., Zhang X.-D., An L.-J., Lv H.-G., He J., Gao C.-J., Sun X.-D. (2012). High-Mobility Group Box 1 Contributes to Mechanical Allodynia and Spinal Astrocytic Activation in a Mouse Model of Type 2 Diabetes. Brain Res. Bull..

[38] Tong W., Wang W., Huang J., Ren N., Wu S.-X., Li Y.-Q. (2010). Spinal High-Mobility Group Box 1 Contributes to Mechanical Allodynia in a Rat Model of Bone Cancer Pain. Biochem. Biophys. Res. Commun..

[39] Shibasaki M., Sasaki M., Miura M., Mizukoshi K., Ueno H., Hashimoto S., Tanaka Y., Amaya F. (2010). Induction of High Mobility Group Box-1 in Dorsal Root Ganglion Contributes to Pain Hypersensitivity after Peripheral Nerve Injury. Pain.

[40] Nakamura Y., Morioka N., Abe H., Zhang F.F., Hisaoka-Nakashima K., Liu K., Nishibori M., Nakata Y. (2013). Neuropathic Pain in Rats with a Partial Sciatic Nerve Ligation Is Alleviated by Intravenous Injection of Monoclonal Antibody to High Mobility Group Box-1. PLoS ONE.

[41] Kochi T., Nakamura Y., Ma S., Hisaoka-Nakashima K., Wang D., Liu K., Wake H., Nishibori M., Irifune M., Morioka N. (2021). Pretreatment with High Mobility Group Box-1 Monoclonal Antibody Prevents the Onset of Trigeminal Neuropathy in Mice with a Distal Infraorbital Nerve Chronic Constriction Injury. Molecules.

[42] Guo L.-H., Schluesener H.J. (2007). The Innate Immunity of the Central Nervous System in Chronic Pain: The Role of Toll-like Receptors. Cell. Mol. Life Sci..

[43] Buehlmann D., Ielacqua G.D., Xandry J., Rudin M. (2019). Prospective Administration of Anti-Nerve Growth Factor Treatment Effectively Suppresses Functional Connectivity Alterations after Cancer-Induced Bone Pain in Mice. Pain.

[44] Lotze M.T., Tracey K.J. (2005). High-Mobility Group Box 1 Protein (HMGB1): Nuclear Weapon in the Immune Arsenal. Nat. Rev. Immunol..

[45] Yu S.P.-C., Hunter D.J. (2015). Emerging Drugs for the Treatment of Knee Osteoarthritis. Expert Opin. Emerg. Drugs.

[46] Hunter D.J., Bierma-Zeinstra S. (2019). Osteoarthritis. Lancet.

[47] Schmelz M., Mantyh P., Malfait A.-M., Farrar J., Yaksh T., Tive L., Viktrup L. (2019). Nerve Growth Factor Antibody for the Treatment of Osteoarthritis Pain and Chronic Low-Back Pain: Mechanism of Action in the Context of Efficacy and Safety. Pain.

[48] Blikman T., Rienstra W., van Raay J.J.A.M., Dijkstra B., Bulstra S.K., Stevens M., van den Akker-Scheek I. (2018). Neuropathic-like Symptoms and the Association with Joint-Specific Function and Quality of Life in Patients with Hip and Knee Osteoarthritis. PLoS ONE.

[49] Berenbaum F., Blanco F.J., Guermazi A., Miki K., Yamabe T., Viktrup L., Junor R., Carey W., Brown M.T., West C.R. (2020). Subcutaneous Tanezumab for Osteoarthritis of the Hip or Knee: Efficacy and Safety Results from a 24-Week Randomised Phase III Study with a 24-Week Follow-up Period. Ann. Rheum. Dis..

[50] Cai X., Yuan S., Zeng Y., Wang C., Yu N., Ding C. (2021). New Trends in Pharmacological Treatments for Osteoarthritis. Front. Pharmacol..

[51] Wise B.L., Seidel M.F., Lane N.E. (2021). The Evolution of Nerve Growth Factor Inhibition in Clinical Medicine. Nat. Rev. Rheumatol..

[52] Ashraf S., Mapp P.I., Burston J., Bennett A.J., Chapman V., Walsh D.A. (2014). Augmented Pain Behavioural Responses to Intra-Articular Injection of Nerve Growth Factor in Two Animal Models of Osteoarthritis. Ann. Rheum. Dis..

[53] Hoshino T., Tsuji K., Onuma H., Udo M., Ueki H., Akiyama M., Abula K., Katagiri H., Miyatake K., Watanabe T. (2018). Persistent Synovial Inflammation Plays Important Roles in Persistent Pain Development in the Rat Knee before Cartilage Degradation Reaches the Subchondral Bone. BMC Musculoskelet. Disord..

[54] Zhu S., Zhu J., Zhen G., Hu Y., An S., Li Y., Zheng Q., Chen Z., Yang Y., Wan M. (2019). Subchondral Bone Osteoclasts Induce Sensory Innervation and Osteoarthritis Pain. J. Clin. Investig..

[55] Hong J.-I., Park I.Y., Kim H.A. (2020). Understanding the Molecular Mechanisms Underlying the Pathogenesis of Arthritis Pain Using Animal Models. Int. J. Mol. Sci..

[56] McNamee K.E., Burleigh A., Gompels L.L., Feldmann M., Allen S.J., Williams R.O., Dawbarn D., Vincent T.L., Inglis J.J. (2010). Treatment of Murine Osteoarthritis with TrkAd5 Reveals a Pivotal Role for Nerve Growth Factor in Non-Inflammatory Joint Pain. Pain.

[57] Kc R., Li X., Kroin J.S., Liu Z., Chen D., Xiao G., Levine B., Li J., Hamilton J.L., van Wijnen A.J. (2016). PKCδ Null Mutations in a Mouse Model of Osteoarthritis Alter Osteoarthritic Pain Independently of Joint Pathology by Augmenting NGF/TrkA-Induced Axonal Outgrowth. Ann. Rheum. Dis..

[58] LaBranche T.P., Bendele A.M., Omura B.C., Gropp K.E., Hurst S.I., Bagi C.M., Cummings T.R., Grantham L.E., Shelton D.L., Zorbas M.A. (2017). Nerve Growth Factor Inhibition with Tanezumab Influences Weight-Bearing and Subsequent Cartilage Damage in the Rat Medial Meniscal Tear Model. Ann. Rheum. Dis..

[59] Majuta L.A., Guedon J.-M.G., Mitchell S.A.T., Ossipov M.H., Mantyh P.W. (2017). Anti-Nerve Growth Factor Therapy Increases Spontaneous Day/Night Activity in Mice with Orthopedic Surgery-Induced Pain. Pain.

[60] Flannery C.R., Moran N., Blasioli D., Donahue K., Kane J., Gladysheva T., Dagher R., Fang R., Vardanyan A., Bangari D. (2015). Efficacy of a Novel, Locally Delivered TrkA Inhibitor in Preclinical Models of OA and Joint Pain. Osteoarthr. Cartil..

[61] Ishikawa G., Koya Y., Tanaka H., Nagakura Y. (2015). Long-Term Analgesic Effect of a Single Dose of Anti-NGF Antibody on Pain during Motion without Notable Suppression of Joint Edema and Lesion in a Rat Model of Osteoarthritis. Osteoarthr. Cartil..

[62] Bryden L.A., Nicholson J.R., Doods H., Pekcec A. (2015). Deficits in Spontaneous Burrowing Behavior in the Rat Bilateral Monosodium Iodoacetate Model of Osteoarthritis: An Objective Measure of Pain-Related Behavior and Analgesic Efficacy. Osteoarthr. Cartil..

[63] Xu L., Nwosu L.N., Burston J.J., Millns P.J., Sagar D.R., Mapp P.I., Meesawatsom P., Li L., Bennett A.J., Walsh D.A. (2016). The Anti-NGF Antibody MuMab 911 Both Prevents and Reverses Pain Behaviour and Subchondral Osteoclast Numbers in a Rat Model of Osteoarthritis Pain. Osteoarthr. Cartil..

[64] Miyagi M., Ishikawa T., Kamoda H., Suzuki M., Inoue G., Sakuma Y., Oikawa Y., Orita S., Uchida K., Takahashi K. (2017). Efficacy of Nerve Growth Factor Antibody in a Knee Osteoarthritis Pain Model in Mice. BMC Musculoskelet. Disord..

[65] Larkin J., Lohr T.A., Elefante L., Shearin J., Matico R., Su J.-L., Xue Y., Liu F., Genell C., Miller R.E. (2015). Translational Development of an ADAMTS-5 Antibody for Osteoarthritis Disease Modification. Osteoarthr. Cartil..

[66] Nwosu L.N., Mapp P.I., Chapman V., Walsh D.A. (2016). Blocking the Tropomyosin Receptor Kinase A (TrkA) Receptor Inhibits Pain Behaviour in Two Rat Models of Osteoarthritis. Ann. Rheum. Dis..

[67] Lascelles B.D.X., Knazovicky D., Case B., Freire M., Innes J.F., Drew A.C., Gearing D.P. (2015). A Canine-Specific Anti-Nerve Growth Factor Antibody Alleviates Pain and Improves Mobility and Function in Dogs with Degenerative Joint Disease-Associated Pain. BMC Vet. Res..

[68] Brown M.T., Murphy F.T., Radin D.M., Davignon I., Smith M.D., West C.R. (2012). Tanezumab Reduces Osteoarthritic Knee Pain: Results of a Randomized, Double-Blind, Placebo-Controlled Phase III Trial. J. Pain.

[69] Ekman E.F., Gimbel J.S., Bello A.E., Smith M.D., Keller D.S., Annis K.M., Brown M.T., West C.R., Verburg K.M. (2014). Efficacy and Safety of Intravenous Tanezumab for the Symptomatic Treatment of Osteoarthritis: 2 Randomized Controlled Trials versus Naproxen. J. Rheumatol..

[70] Birbara C., Dabezies E.J., Burr A.M., Fountaine R.J., Smith M.D., Brown M.T., West C.R., Arends R.H., Verburg K.M. (2018). Safety and Efficacy of Subcutaneous Tanezumab in Patients with Knee or Hip Osteoarthritis. J. Pain Res..

[71] Schnitzer T.J., Khan A., Bessette L., Davignon I., Brown M.T., Pixton G., Prucka W.R., Tive L., Viktrup L., West C.R. (2020). Onset and Maintenance of Efficacy of Subcutaneous Tanezumab in Patients with Moderate to Severe Osteoarthritis of the Knee or Hip: A 16-Week Dose-Titration Study. Semin. Arthritis Rheum..

[72] Tive L., Bello A.E., Radin D., Schnitzer T.J., Nguyen H., Brown M.T., West C.R. (2019). Pooled Analysis of Tanezumab Efficacy and Safety with Subgroup Analyses of Phase III Clinical Trials in Patients with Osteoarthritis Pain of the Knee or Hip. J. Pain Res..

[73] Mayorga A.J., Wang S., Kelly K.M., Thipphawong J. (2016). Efficacy and Safety of Fulranumab as Monotherapy in Patients with Moderate to Severe, Chronic Knee Pain of Primary Osteoarthritis: A Randomised, Placebo- and Active-Controlled Trial. Int. J. Clin. Pract..

[74] Sanga P., Katz N., Polverejan E., Wang S., Kelly K.M., Haeussler J., Thipphawong J. (2017). Long-Term Safety and Efficacy of Fulranumab in Patients with Moderate-to-Severe Osteoarthritis Pain: A Phase II Randomized, Double-Blind, Placebo-Controlled Extension Study. Arthritis Rheumatol..

[75] Dakin P., DiMartino S.J., Gao H., Maloney J., Kivitz A.J., Schnitzer T.J., Stahl N., Yancopoulos G.D., Geba G.P. (2019). The Efficacy, Tolerability, and Joint Safety of Fasinumab in Osteoarthritis Pain: A Phase IIb/III Double-Blind, Placebo-Controlled, Randomized Clinical Trial. Arthritis Rheumatol..

[76] Balanescu A.R., Feist E., Wolfram G., Davignon I., Smith M.D., Brown M.T., West C.R. (2014). Efficacy and Safety of Tanezumab Added on to Diclofenac Sustained Release in Patients with Knee or Hip Osteoarthritis: A Double-Blind, Placebo-Controlled, Parallel-Group, Multicentre Phase III Randomised Clinical Trial. Ann. Rheum. Dis..

[77] Schnitzer T.J., Ekman E.F., Spierings E.L.H., Greenberg H.S., Smith M.D., Brown M.T., West C.R., Verburg K.M. (2015). Efficacy and Safety of Tanezumab Monotherapy or Combined with Non-Steroidal Anti-Inflammatory Drugs in the Treatment of Knee or Hip Osteoarthritis Pain. Ann. Rheum. Dis..

[78] Hochberg M.C., Carrino J.A., Schnitzer T.J., Guermazi A., Walsh D.A., White A., Nakajo S., Fountaine R.J., Hickman A., Pixton G. (2021). Long-Term Safety and Efficacy of Subcutaneous Tanezumab Versus Nonsteroidal Antiinflammatory Drugs for Hip or Knee Osteoarthritis: A Randomized Trial. Arthritis Rheumatol..

[79] Spierings E.L.H., Fidelholtz J., Wolfram G., Smith M.D., Brown M.T., West C.R. (2013). A Phase III Placebo- and Oxycodone-Controlled Study of Tanezumab in Adults with Osteoarthritis Pain of the Hip or Knee. Pain.

[80] Brown M.T., Murphy F.T., Radin D.M., Davignon I., Smith M.D., West C.R. (2013). Tanezumab Reduces Osteoarthritic Hip Pain: Results of a Randomized, Double-Blind, Placebo-Controlled Phase III Trial. Arthritis Rheum..

[81] Lane N.E., Schnitzer T.J., Birbara C.A., Mokhtarani M., Shelton D.L., Smith M.D., Brown M.T. (2010). Tanezumab for the Treatment of Pain from Osteoarthritis of the Knee. N. Engl. J. Med..

[82] Berenbaum F., Langford R., Perrot S., Miki K., Blanco F.J., Yamabe T., Isogawa N., Junor R., Carey W., Viktrup L. (2021). Subcutaneous Tanezumab for Osteoarthritis: Is the Early Improvement in Pain and Function Meaningful and Sustained?. Eur. J. Pain.

[83] Schnitzer T.J., Easton R., Pang S., Levinson D.J., Pixton G., Viktrup L., Davignon I., Brown M.T., West C.R., Verburg K.M. (2019). Effect of Tanezumab on Joint Pain, Physical Function, and Patient Global Assessment of Osteoarthritis Among Patients with Osteoarthritis of the Hip or Knee: A Randomized Clinical Trial. JAMA.

[84] Bimonte S., Cascella M., Forte C.A., Esposito G., Cuomo A. (2021). The Role of Anti-Nerve Growth Factor Monoclonal Antibodies in the Control of Chronic Cancer and Non-Cancer Pain. J. Pain Res..

[85] Sparks J.A. (2019). Rheumatoid Arthritis. Ann. Intern. Med..

[86] Atzeni F., Nucera V., Masala I.F., Sarzi-Puttini P., Bonitta G. (2019). Il-6 Involvement in Pain, Fatigue and Mood Disorders in Rheumatoid Arthritis and the Effects of Il-6 Inhibitor Sarilumab. Pharmacol. Res..

[87] Crotti C., Biggioggero M., Becciolini A., Favalli E.G. (2018). Sarilumab: Patient-Reported Outcomes in Rheumatoid Arthritis. Patient Relat. Outcome Meas..

[88] Fonseca J.E., Santos M.J., Canhão H., Choy E. (2009). Interleukin-6 as a Key Player in Systemic Inflammation and Joint Destruction. Autoimmun. Rev..

[89] Song S.-N.J., Tomosugi N., Kawabata H., Ishikawa T., Nishikawa T., Yoshizaki K. (2010). Down-Regulation of Hepcidin Resulting from Long-Term Treatment with an Anti-IL-6 Receptor Antibody (Tocilizumab) Improves Anemia of Inflammation in Multicentric Castleman Disease. Blood.

[90] Bullock J., Rizvi S.A.A., Saleh A.M., Ahmed S.S., Do D.P., Ansari R.A., Ahmed J. (2019). Rheumatoid Arthritis: A Brief Overview of the Treatment. Med. Princ. Pract..

[91] Mahmood Z., Schmalzing M., Dörner T., Tony H.-P., Muhammad K. (2020). Therapeutic Cytokine Inhibition Modulates Activation and Homing Receptors of Peripheral Memory B Cell Subsets in Rheumatoid Arthritis Patients. Front. Immunol..

[92] Zhang A., Lee Y.C. (2018). Mechanisms for Joint Pain in Rheumatoid Arthritis (RA): From Cytokines to Central Sensitization. Curr. Osteoporos. Rep..

[93] Lee H., Lee M.-Y., Bhang S.H., Kim B.-S., Kim Y.S., Ju J.H., Kim K.S., Hahn S.K. (2014). Hyaluronate-Gold Nanoparticle/Tocilizumab Complex for the Treatment of Rheumatoid Arthritis. ACS Nano.

[94] Monnet E., Shang L., Lapeyre G., deGraaf K., Hatterer E., Buatois V., Elson G., Ferlin W., Gabay C., Sokolove J. (2015). AB0451 NI-0101, a Monoclonal Antibody Targeting Toll Like Receptor 4 (TLR4) Being Developed for Rheumatoid Arthritis (RA) Treatment with a Potential for Personalized Medicine. Ann. Rheum. Dis..

[95] Ashraf S., Bouhana K.S., Pheneger J., Andrews S.W., Walsh D.A. (2016). Selective Inhibition of Tropomyosin-Receptor-Kinase A (TrkA) Reduces Pain and Joint Damage in Two Rat Models of Inflammatory Arthritis. Arthritis Res. Ther..

[96] Segond von Banchet G., König C., Patzer J., Eitner A., Leuchtweis J., Ebbinghaus M., Boettger M.K., Schaible H.-G. (2016). Long-Lasting Activation of the Transcription Factor CREB in Sensory Neurons by Interleukin-1β During Antigen-Induced Arthritis in Rats: A Mechanism of Persistent Arthritis Pain?. Arthritis Rheumatol..

[97] Bonetti N.R., Diaz-Cañestro C., Liberale L., Crucet M., Akhmedov A., Merlini M., Reiner M.F., Gobbato S., Stivala S., Kollias G. (2019). Tumour Necrosis Factor-α Inhibition Improves Stroke Outcome in a Mouse Model of Rheumatoid Arthritis. Sci. Rep..

[98] Makalish T.P., Golovkin I.O., Oberemok V.V., Laikova K.V., Temirova Z.Z., Serdyukova O.A., Novikov I.A., Rosovskyi R.A., Gordienko A.I., Zyablitskaya E.Y. (2021). Anti-Rheumatic Effect of Antisense Oligonucleotide Cytos-11 Targeting TNF-α Expression. Int. J. Mol. Sci..

[99] Lee Y.-A., Hahm D.-H., Kim J.Y., Sur B., Lee H.M., Ryu C.J., Yang H.-I., Kim K.S. (2018). Potential Therapeutic Antibodies Targeting Specific Adiponectin Isoforms in Rheumatoid Arthritis. Arthritis Res. Ther..

[100] Wang L., Jiang X., Zheng Q., Jeon S.-M., Chen T., Liu Y., Kulaga H., Reed R., Dong X., Caterina M.J. (2019). Neuronal FcγRI Mediates Acute and Chronic Joint Pain. J. Clin. Investig..

[101] Lamb Y.N., Deeks E.D. (2018). Sarilumab: A Review in Moderate to Severe Rheumatoid Arthritis. Drugs.

[102] Chen Y.-F., Jobanputra P., Barton P., Jowett S., Bryan S., Clark W., Fry-Smith A., Burls A. (2006). A Systematic Review of the Effectiveness of Adalimumab, Etanercept and Infliximab for the Treatment of Rheumatoid Arthritis in Adults and an Economic Evaluation of Their Cost-Effectiveness. Health Technol. Assess..

[103] Smolen J.S., Beaulieu A., Rubbert-Roth A., Ramos-Remus C., Rovensky J., Alecock E., Woodworth T., Alten R. (2008). OPTION Investigators Effect of Interleukin-6 Receptor Inhibition with Tocilizumab in Patients with Rheumatoid Arthritis (OPTION Study): A Double-Blind, Placebo-Controlled, Randomised Trial. Lancet.

[104] Kremer J.M., Blanco R., Brzosko M., Burgos-Vargas R., Halland A.-M., Vernon E., Ambs P., Fleischmann R. (2011). Tocilizumab Inhibits Structural Joint Damage in Rheumatoid Arthritis Patients with Inadequate Responses to Methotrexate: Results from the Double-Blind Treatment Phase of a Randomized Placebo-Controlled Trial of Tocilizumab Safety and Prevention of Structural Joint Damage at One Year. Arthritis Rheum..

[105] Fleischmann R.M., Halland A.-M., Brzosko M., Burgos-Vargas R., Mela C., Vernon E., Kremer J.M. (2013). Tocilizumab Inhibits Structural Joint Damage and Improves Physical Function in Patients with Rheumatoid Arthritis and Inadequate Responses to Methotrexate: LITHE Study 2-Year Results. J. Rheumatol..

[106] Almeida C., Choy E.H.S., Hewlett S., Kirwan J.R., Cramp F., Chalder T., Pollock J., Christensen R. (2016). Biologic Interventions for Fatigue in Rheumatoid Arthritis. Cochrane Database Syst. Rev..

[107] Tumor Necrosis Factor Antagonists (2012). LiverTox: Clinical and Research Information on Drug-Induced Liver Injury.

[108] Mok C.C. (2013). Rituximab for the Treatment of Rheumatoid Arthritis: An Update. Drug Des. Devel. Ther..

[109] Sacco S., Bendtsen L., Ashina M., Reuter U., Terwindt G., Mitsikostas D.-D., Martelletti P. (2019). European Headache Federation Guideline on the Use of Monoclonal Antibodies Acting on the Calcitonin Gene Related Peptide or Its Receptor for Migraine Prevention. J. Headache Pain.

[110] Charles A., Pozo-Rosich P. (2019). Targeting Calcitonin Gene-Related Peptide: A New Era in Migraine Therapy. Lancet.

[111] Yuan H., Lauritsen C.G., Kaiser E.A., Silberstein S.D. (2017). CGRP Monoclonal Antibodies for Migraine: Rationale and Progress. BioDrugs.

[112] Hansen J.M., Hauge A.W., Olesen J., Ashina M. (2010). Calcitonin Gene-Related Peptide Triggers Migraine-like Attacks in Patients with Migraine with Aura. Cephalalgia.

[113] Raddant A.C., Russo A.F. (2011). Calcitonin Gene-Related Peptide in Migraine: Intersection of Peripheral Inflammation and Central Modulation. Expert Rev. Mol. Med..

[114] Iyengar S., Ossipov M.H., Johnson K.W. (2017). The Role of Calcitonin Gene-Related Peptide in Peripheral and Central Pain Mechanisms Including Migraine. Pain.

[115] Wattiez A.-S., Wang M., Russo A.F. (2019). CGRP in Animal Models of Migraine. Handb. Exp. Pharmacol..

[116] Mason B.N., Kaiser E.A., Kuburas A., Loomis M.-C.M., Latham J.A., Garcia-Martinez L.F., Russo A.F. (2017). Induction of Migraine-Like Photophobic Behavior in Mice by Both Peripheral and Central CGRP Mechanisms. J. Neurosci..

[117] Rea B.J., Wattiez A.-S., Waite J.S., Castonguay W.C., Schmidt C.M., Fairbanks A.M., Robertson B.R., Brown C.J., Mason B.N., Moldovan-Loomis M.-C. (2018). Peripherally Administered Calcitonin Gene-Related Peptide Induces Spontaneous Pain in Mice: Implications for Migraine. Pain.

[118] Wattiez A.-S., Sowers L.P., Russo A.F. (2020). Calcitonin Gene-Related Peptide (CGRP): Role in Migraine Pathophysiology and Therapeutic Targeting. Expert Opin. Ther. Targets.

[119] Dodick D.W., Ashina M., Brandes J.L., Kudrow D., Lanteri-Minet M., Osipova V., Palmer K., Picard H., Mikol D.D., Lenz R.A. (2018). ARISE: A Phase 3 Randomized Trial of Erenumab for Episodic Migraine. Cephalalgia.

[120] Sun H., Dodick D.W., Silberstein S., Goadsby P.J., Reuter U., Ashina M., Saper J., Cady R., Chon Y., Dietrich J. (2016). Safety and Efficacy of AMG 334 for Prevention of Episodic Migraine: A Randomised, Double-Blind, Placebo-Controlled, Phase 2 Trial. Lancet Neurol..

[121] Ashina M., Saper J., Cady R., Schaeffler B.A., Biondi D.M., Hirman J., Pederson S., Allan B., Smith J. (2020). Eptinezumab in Episodic Migraine: A Randomized, Double-Blind, Placebo-Controlled Study (PROMISE-1). Cephalalgia.

[122] Smith T.R., Janelidze M., Chakhava G., Cady R., Hirman J., Allan B., Pederson S., Smith J., Schaeffler B. (2020). Eptinezumab for the Prevention of Episodic Migraine: Sustained Effect Through 1 Year of Treatment in the PROMISE-1 Study. Clin. Ther..

[123] Tepper S., Ashina M., Reuter U., Brandes J.L., Doležil D., Silberstein S., Winner P., Leonardi D., Mikol D., Lenz R. (2017). Safety and Efficacy of Erenumab for Preventive Treatment of Chronic Migraine: A Randomised, Double-Blind, Placebo-Controlled Phase 2 Trial. Lancet Neurol..

[124] Lipton R.B., Goadsby P.J., Smith J., Schaeffler B.A., Biondi D.M., Hirman J., Pederson S., Allan B., Cady R. (2020). Efficacy and Safety of Eptinezumab in Patients with Chronic Migraine: PROMISE-2. Neurology.

[125] Bigal M.E., Edvinsson L., Rapoport A.M., Lipton R.B., Spierings E.L.H., Diener H.-C., Burstein R., Loupe P.S., Ma Y., Yang R. (2015). Safety, Tolerability, and Efficacy of TEV-48125 for Preventive Treatment of Chronic Migraine: A Multicentre, Randomised, Double-Blind, Placebo-Controlled, Phase 2b Study. Lancet Neurol..

[126] Silberstein S., Diamond M., Hindiyeh N.A., Biondi D.M., Cady R., Hirman J., Allan B., Pederson S., Schaeffler B., Smith J. (2020). Eptinezumab for the Prevention of Chronic Migraine: Efficacy and Safety through 24 Weeks of Treatment in the Phase 3 PROMISE-2 (Prevention of Migraine via Intravenous ALD403 Safety and Efficacy-2) Study. J. Headache Pain.

[127] Villar-Martínez M.D., Moreno-Ajona D., Goadsby P.J. (2021). Eptinezumab for the Preventive Treatment of Migraine. Pain Manag..

[128] Dimitroulas T., Lambe T., Raphael J.H., Kitas G.D., Duarte R.V. (2019). Biologic Drugs as Analgesics for the Management of Low Back Pain and Sciatica. Pain Med..

[129] Williams N.H., Lewis R., Din N.U., Matar H.E., Fitzsimmons D., Phillips C.J., Sutton A., Burton K., Hendry M., Nafees S. (2013). A Systematic Review and Meta-Analysis of Biological Treatments Targeting Tumour Necrosis Factor α for Sciatica. Eur. Spine J..

[130] Wang Y.F., Chen P.Y., Chang W., Zhu F.Q., Xu L.L., Wang S.L., Chang L.Y., Luo J., Liu G.J. (2014). Clinical Significance of Tumor Necrosis Factor-α Inhibitors in the Treatment of Sciatica: A Systematic Review and Meta-Analysis. PLoS ONE.

[131] Leite V.F., Buehler A.M., El Abd O., Benyamin R.M., Pimentel D.C., Chen J., Hsing W.T., Mazloomdoost D., Amadera J.E.D. (2014). Anti-Nerve Growth Factor in the Treatment of Low Back Pain and Radiculopathy: A Systematic Review and a Meta-Analysis. Pain Physician.

[132] Markman J.D., Bolash R.B., McAlindon T.E., Kivitz A.J., Pombo-Suarez M., Ohtori S., Roemer F.W., Li D.J., Viktrup L., Bramson C. (2020). Tanezumab for Chronic Low Back Pain: A Randomized, Double-Blind, Placebo- and Active-Controlled, Phase 3 Study of Efficacy and Safety. Pain.

[133] Kivitz A.J., Gimbel J.S., Bramson C., Nemeth M.A., Keller D.S., Brown M.T., West C.R., Verburg K.M. (2013). Efficacy and Safety of Tanezumab versus Naproxen in the Treatment of Chronic Low Back Pain. Pain.

[134] Gimbel J.S., Kivitz A.J., Bramson C., Nemeth M.A., Keller D.S., Brown M.T., West C.R., Verburg K.M. (2014). Long-Term Safety and Effectiveness of Tanezumab as Treatment for Chronic Low Back Pain. Pain.

[135] Montague K., Malcangio M. (2017). The Therapeutic Potential of Monocyte/Macrophage Manipulation in the Treatment of Chemotherapy-Induced Painful Neuropathy. Front. Mol. Neurosci..

[136] Cheng H.T., Dauch J.R., Hayes J.M., Yanik B.M., Feldman E.L. (2012). Nerve Growth Factor/P38 Signaling Increases Intraepidermal Nerve Fiber Densities in Painful Neuropathy of Type 2 Diabetes. Neurobiol. Dis..

[137] Mathews J.A., Krishnamoorthy N., Kasahara D.I., Hutchinson J., Cho Y., Brand J.D., Williams A.S., Wurmbrand A.P., Ribeiro L., Cuttitta F. (2018). Augmented Responses to Ozone in Obese Mice Require IL-17A and Gastrin-Releasing Peptide. Am. J. Respir. Cell Mol. Biol..

[138] Banach M., Juranek J.K., Zygulska A.L. (2017). Chemotherapy-Induced Neuropathies-a Growing Problem for Patients and Health Care Providers. Brain Behav..

[139] Hu L.-Y., Mi W.-L., Wu G.-C., Wang Y.-Q., Mao-Ying Q.-L. (2019). Prevention and Treatment for Chemotherapy-Induced Peripheral Neuropathy: Therapies Based on CIPN Mechanisms. Curr. Neuropharmacol..

[140] Griffiths L.A., Duggett N.A., Pitcher A.L., Flatters S.J.L. (2018). Evoked and Ongoing Pain-Like Behaviours in a Rat Model of Paclitaxel-Induced Peripheral Neuropathy. Pain Res. Manag..

[141] Kiguchi N., Kobayashi Y., Maeda T., Fukazawa Y., Tohya K., Kimura M., Kishioka S. (2012). Epigenetic Augmentation of the Macrophage Inflammatory Protein 2/C-X-C Chemokine Receptor Type 2 Axis through Histone H3 Acetylation in Injured Peripheral Nerves Elicits Neuropathic Pain. J. Pharmacol. Exp. Ther..

[142] Wild K.D., Bian D., Zhu D., Davis J., Bannon A.W., Zhang T.J., Louis J.-C. (2007). Antibodies to Nerve Growth Factor Reverse Established Tactile Allodynia in Rodent Models of Neuropathic Pain without Tolerance. J. Pharmacol. Exp. Ther..

[143] Gwak Y.S., Nam T.S., Paik K.S., Hulsebosch C.E., Leem J.W. (2003). Attenuation of Mechanical Hyperalgesia Following Spinal Cord Injury by Administration of Antibodies to Nerve Growth Factor in the Rat. Neurosci. Lett..

[144] Pfizer (2021). A Phase II Randomized, Double-Blind, Placebo-Controlled, Multicenter, Parallel Group, Proof of Concept Study of the Analgesic Effects of rn624 in Adult Patients with Post-Herpetic Neuralgia. https://clinicaltrials.gov/ct2/show/NCT00568321.

[145] Bramson C., Herrmann D.N., Carey W., Keller D., Brown M.T., West C.R., Verburg K.M., Dyck P.J. (2015). Exploring the Role of Tanezumab as a Novel Treatment for the Relief of Neuropathic Pain. Pain Med..

[146] NCIC Clinical Trials Group (2012). A Multi-Centre Phase II Trial Investigating the Efficacy and Tolerability of Bortezomib Added to Cyclophosphamide, Vincristine, Prednisone, and Rituximab (BCVP-R) for Patients with Advanced Stage Follicular Non-Hodgkin’s Lymphoma Requiring Systemic First-Line Treatment. https://clinicaltrials.gov/ct2/show/NCT00428142.

[147] Northwestern University (2019). A Phase I/II Trial of Combined Weekly Bortezomib (VELCADE®) and Y-90-Ibritumomab Tiuxetan (Zevalin) in Patients with Relapsed or Refractory Follicular Lymphoma and Transformed Non-Hodgkin’s Lymphoma. https://clinicaltrials.gov/ct2/show/NCT00372905.

[148] KU (2008). Leuven Anti TNFa Treatment for Deep Endometriosis Associated Pain: A Randomised Placebo Controlled Trial. https://clinicaltrials.gov/ct2/show/NCT00604864.

[149] Koninckx P.R., Craessaerts M., Timmerman D., Cornillie F., Kennedy S. (2008). Anti-TNF-α Treatment for Deep Endometriosis-Associated Pain: A Randomized Placebo-Controlled Trial. Hum. Reprod..

[150] Teva Branded Pharmaceutical Products R & D, Inc (2021). A Multicenter, Randomized, Double-Blind, Placebo-Controlled, Proof of Concept Study of the Efficacy and Safety of Fremanezumab for Treatment of Patients with Fibromyalgia. https://clinicaltrials.gov/ct2/show/NCT03965091.

[151] Jimenez-Andrade J.M., Ghilardi J.R., Castañeda-Corral G., Kuskowski M.A., Mantyh P.W. (2011). Preventive or Late Administration of Anti-NGF Therapy Attenuates Tumor-Induced Nerve Sprouting, Neuroma Formation, and Cancer Pain. Pain.

[152] Currie G.L., Delaney A., Bennett M.I., Dickenson A.H., Egan K.J., Vesterinen H.M., Sena E.S., Macleod M.R., Colvin L.A., Fallon M.T. (2013). Animal Models of Bone Cancer Pain: Systematic Review and Meta-Analyses. Pain.

[153] Mantyh P.W., Koltzenburg M., Mendell L.M., Tive L., Shelton D.L. (2011). Antagonism of Nerve Growth Factor-TrkA Signaling and the Relief of Pain. Anesthesiology.

[154] Sevcik M.A., Ghilardi J.R., Peters C.M., Lindsay T.H., Halvorson K.G., Jonas B.M., Kubota K., Kuskowski M.A., Boustany L., Shelton D.L. (2005). Anti-NGF Therapy Profoundly Reduces Bone Cancer Pain and the Accompanying Increase in Markers of Peripheral and Central Sensitization. Pain.

[155] Majuta L.A., Mitchell S.A.T., Kuskowski M.A., Mantyh P.W. (2018). Anti-Nerve Growth Factor Does Not Change Physical Activity in Normal Young or Aging Mice but Does Increase Activity in Mice with Skeletal Pain. Pain.

[156] Almholt K., Hebsgaard J.B., Nansen A., Andersson C., Pass J., Rønø B., Thygesen P., Pelzer H., Loftager M., Lund I.K. (2018). Antibody-Mediated Neutralization of UPA Proteolytic Function Reduces Disease Progression in Mouse Arthritis Models. J. Immunol..

[157] Hu R., Song Y.-F., Yang Z.-Y., Zhang C., Tan B. (2021). Clinical Outcomes of Tanezumab with Different Dosages for Patient with Osteoarthritis: Network Meta-Analysis. Front. Pharmacol..

[158] Gjefsen E., Bråten L.C.H., Goll G.L., Wigemyr M., Bolstad N., Valberg M., Schistad E.I., Marchand G.H., Granviken F., Selmer K.K. (2020). The Effect of Infliximab in Patients with Chronic Low Back Pain and Modic Changes (the BackToBasic Study): Study Protocol of a Randomized, Double Blind, Placebo-Controlled, Multicenter Trial. BMC Musculoskelet. Disord..

[159] Tepper S.J., Diener H.-C., Ashina M., Brandes J.L., Friedman D.I., Reuter U., Cheng S., Nilsen J., Leonardi D.K., Lenz R.A. (2019). Erenumab in Chronic Migraine with Medication Overuse: Subgroup Analysis of a Randomized Trial. Neurology.

[160] Tepper S.J., Ashina M., Reuter U., Brandes J.L., Doležil D., Silberstein S.D., Winner P., Zhang F., Cheng S., Mikol D.D. (2020). Long-Term Safety and Efficacy of Erenumab in Patients with Chronic Migraine: Results from a 52-Week, Open-Label Extension Study. Cephalalgia Int. J. Headache.

[161] Overeem L.H., Neeb L., Reuter U. (2019). Erenumab for Episodic Migraine Prophylaxis. Expert Rev. Neurother..

[162] Garland S.G., Smith S.M., Gums J.G. (2019). Erenumab: A First-in-Class Monoclonal Antibody for Migraine Prevention. Ann. Pharmacother..

[163] Ornello R., Tiseo C., Frattale I., Perrotta G., Marini C., Pistoia F., Sacco S. (2019). The Appropriate Dosing of Erenumab for Migraine Prevention after Multiple Preventive Treatment Failures: A Critical Appraisal. J. Headache Pain.

[164] Ashina M., Cohen J.M., Gandhi S.K., Du E. (2021). Reduction in the Severity and Duration of Headache Following Fremanezumab Treatment in Patients with Episodic and Chronic Migraine. Headache.

[165] Cohen J.M., Dodick D.W., Yang R., Newman L.C., Li T., Aycardi E., Bigal M.E. (2017). Fremanezumab as Add-On Treatment for Patients Treated with Other Migraine Preventive Medicines. Headache.

[166] Halker Singh R.B., Aycardi E., Bigal M.E., Loupe P.S., McDonald M., Dodick D.W. (2019). Sustained Reductions in Migraine Days, Moderate-to-Severe Headache Days and Days with Acute Medication Use for HFEM and CM Patients Taking Fremanezumab: Post-Hoc Analyses from Phase 2 Trials. Cephalalgia.

[167] Silberstein S.D., Rapoport A.M., Loupe P.S., Aycardi E., McDonald M., Yang R., Bigal M.E. (2019). The Effect of Beginning Treatment with Fremanezumab on Headache and Associated Symptoms in the Randomized Phase 2 Study of High Frequency Episodic Migraine: Post-Hoc Analyses on the First 3 Weeks of Treatment. Headache.

[168] Silberstein S.D., Cohen J.M., Yang R., Gandhi S.K., Du E., Jann A.E., Marmura M.J. (2021). Treatment Benefit among Migraine Patients Taking Fremanezumab: Results from a Post Hoc Responder Analysis of Two Placebo-Controlled Trials. J. Headache Pain.

[169] Martin V., Samaan K.H., Aurora S., Pearlman E.M., Zhou C., Li X., Pallay R. (2020). Efficacy and Safety of Galcanezumab for the Preventive Treatment of Migraine: A Narrative Review. Adv. Ther..

[170] Dodick D.W., Goadsby P.J., Spierings E.L.H., Scherer J.C., Sweeney S.P., Grayzel D.S. (2014). Safety and Efficacy of LY2951742, a Monoclonal Antibody to Calcitonin Gene-Related Peptide, for the Prevention of Migraine: A Phase 2, Randomised, Double-Blind, Placebo-Controlled Study. Lancet Neurol..

[171] Sanofi (2017). A Randomized, Double-Blind, Placebo-Controlled, Multicenter, Two-Part, Dose Ranging and Confirmatory Study with an Operationally Seamless Design, Evaluating Efficacy and Safety of SAR153191 on Top of Methotrexate (MTX) in Patients with Active Rheumatoid Arthritis Who Are Inadequate Responders to MTX Therapy. https://www.clinicaltrials.gov/ct2/show/NCT01061736.

[172] Strand V., Kosinski M., Chen C.-I., Joseph G., Rendas-Baum R., Graham N.M.H., van Hoogstraten H., Bayliss M., Fan C., Huizinga T. (2016). Sarilumab plus Methotrexate Improves Patient-Reported Outcomes in Patients with Active Rheumatoid Arthritis and Inadequate Responses to Methotrexate: Results of a Phase III Trial. Arthritis Res. Ther..

[173] Hoffmann-La Roche (2013). A Randomized, Double-Blind Study of Safety and Prevention of Structural Joint Damage During Treatment with Tocilizumab Versus Placebo, in Combination with Methotrexate, in Patients with Moderate to Severe Rheumatoid Arthritis. https://clinicaltrials.gov/ct2/show/NCT00106535.

[174] Burmester G.R., Landewé R., Genovese M.C., Friedman A.W., Pfeifer N.D., Varothai N.A., Lacerda A.P. (2017). Adalimumab Long-Term Safety: Infections, Vaccination Response and Pregnancy Outcomes in Patients with Rheumatoid Arthritis. Ann. Rheum. Dis..

[175] Fautrel B., Kirkham B., Pope J.E., Takeuchi T., Gaich C., Quebe A., Zhu B., de la Torre I., De Leonardis F., Taylor P.C. (2019). Effect of Baricitinib and Adalimumab in Reducing Pain and Improving Function in Patients with Rheumatoid Arthritis in Low Disease Activity: Exploratory Analyses from RA-BEAM. J. Clin. Med..

[176] Kremer J., Ritchlin C., Mendelsohn A., Baker D., Kim L., Xu Z., Han J., Taylor P. (2010). Golimumab, a New Human Anti-Tumor Necrosis Factor Alpha Antibody, Administered Intravenously in Patients with Active Rheumatoid Arthritis: Forty-Eight-Week Efficacy and Safety Results of a Phase III Randomized, Double-Blind, Placebo-Controlled Study. Arthritis Rheum..

[177] Li Z., Zhang F., Kay J., Fei K., Han C., Zhuang Y., Wu Z., Hsia E.C. (2016). Efficacy and Safety Results from a Phase 3, Randomized, Placebo-Controlled Trial of Subcutaneous Golimumab in Chinese Patients with Active Rheumatoid Arthritis despite Methotrexate Therapy. Int. J. Rheum. Dis..

[178] Ruiz Garcia V., Burls A., Cabello J.B., Vela Casasempere P., Bort-Marti S., Bernal J.A. (2017). Certolizumab Pegol (CDP870) for Rheumatoid Arthritis in Adults. Cochrane Database Syst. Rev..

[179] Bessette L., Haraoui B., Chow A., Fortin I., Dixit S., Khraishi M., Haaland D., Elmoufti S., Staelens F., Bogatyreva I. (2019). Effectiveness and Safety of Certolizumab Pegol in Rheumatoid Arthritis Patients in Canadian Practice: 2-Year Results from the Observational FαsT-CAN Study. Ther. Adv. Musculoskelet. Dis..

[180] Chen W., Li Z., Wang Z., Gao H., Ding J., He Z. (2020). Intraarticular Injection of Infliximab-Loaded Thermosensitive Hydrogel Alleviates Pain and Protects Cartilage in Rheumatoid Arthritis. J. Pain Res..

[181] Thorne C., Bensen W.G., Choquette D., Chow A., Khraishi M., Atkins C.J., Kelsall J.T., Lehman A.J., Shawi M., Khalil H. (2014). Effectiveness and Safety of Infliximab in Rheumatoid Arthritis: Analysis from a Canadian Multicenter Prospective Observational Registry. Arthritis Care Res..

[182] Rigby W., Ferraccioli G., Greenwald M., Zazueta-Montiel B., Fleischmann R., Wassenberg S., Ogale S., Armstrong G., Jahreis A., Burke L. (2011). Effect of Rituximab on Physical Function and Quality of Life in Patients with Rheumatoid Arthritis Previously Untreated with Methotrexate. Arthritis Care Res..

